# Evidence for the Quercetin Binding Site of Glycogen Phosphorylase as a Target for Liver-Isoform-Selective Inhibitors against Glioblastoma: Investigation of Flavanols Epigallocatechin Gallate and Epigallocatechin

**DOI:** 10.1021/acs.jafc.4c06920

**Published:** 2024-10-21

**Authors:** Serafeim Alexopoulos, Megan McGawley, Roshini Mathews, Souzana Papakostopoulou, Symeon Koulas, Demetres D. Leonidas, Tamara Zwain, Joseph M. Hayes, Vasiliki Skamnaki

**Affiliations:** † Department of Biochemistry and Biotechnology, University of Thessaly, Biopolis, Larisa 41500, Greece; ‡ School of Pharmacy & Biomedical Sciences, University of Central Lancashire, Preston PR1 2HE, U.K.

**Keywords:** EGCG, flavanols, glioblastoma, glycogen phosphorylase, quercetin binding site

## Abstract

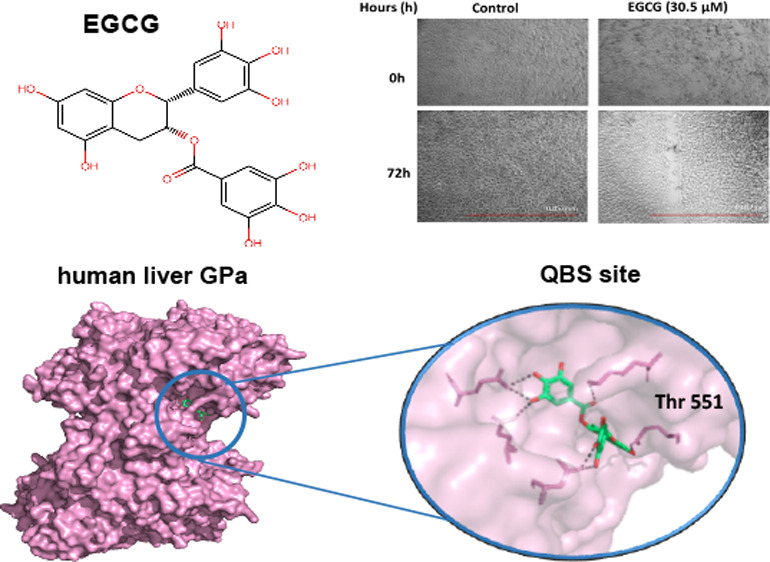

Glycogen phosphorylase (GP) is the rate-determining enzyme in glycogenolysis, and its druggability has been extensively studied over the years for the development of therapeutics against type 2 diabetes (T2D) and, more recently, cancer. However, the conservation of binding sites between the liver and muscle isoforms makes the inhibitor selectivity challenging. Using a combination of kinetic, crystallographic, modeling, and cellular studies, we have probed the binding of dietary flavonoids epigallocatechin gallate (EGCG) and epigallocatechin (EGC) to GP isoforms. The structures of rmGPb-EGCG and rmGPb-EGC complexes were determined by X-ray crystallography, showing binding at the quercetin binding site (QBS) in agreement with kinetic studies that revealed both compounds as noncompetitive inhibitors of GP, with EGCG also causing a significant reduction in cell viability and migration of U87-MG glioblastoma cells. Interestingly, EGCG exhibits different binding modes to GP isoforms, revealing QBS as a promising site for GP targeting, offering new opportunities for the design of liver-selective GP inhibitors.

## Introduction

Inhibition of glycogen degradation (glycogenolysis) in the liver contributes to the control of glycemia and is a long-recognized therapeutic strategy against type 2 diabetes (T2D).^1^ More recently, the role of glycogenolysis in cancer has been recognized, including glioblastoma (GBM).^2,3^ Glycogenolysis occurs through a cascade of hormone-regulated enzyme reactions. Glycogen phosphorylase (GP; EC 2.4.1.1) is the enzyme that catalyzes the first step of glycogenolysis toward the production of glucose-1-phosphate. It is shown that the inhibition of GP is highly effective in reducing hepatic glucose output in rodent models of diabetes, rendering GP a promising molecular target toward the development of antihyperglycemic agents.^4,5^ It was also demonstrated that depleting liver GP through silencing of the hepatic GP isoform gene, the liver isoform was associated with glycogen accumulation, reduced cancer cell proliferation, and corresponding induction of senescence.^6^ A more recent study has additionally shown that liver GP is upregulated in GBM and that glycogen degradation inhibition sensitizes GBM cells to high-dose radiation.^7^ It has also been shown that liver GP is highly expressed in gliomas and that this can be used as a predictive marker for poor prognosis.^8^ Whether the use of GP inhibitors, rather than modulation of liver GP isoform expression, could be beneficial for the treatment of cancer is under investigation. On this basis, a combined direct study on GP inhibitor design for GBM was reported, with promising anticancer effects demonstrated for baicalein.^9^ GP exists in 3 main tissue-specific isoforms in the liver, muscle, and brain. GP is an allosteric enzyme according to the Wyman–Monod–Changeux model and, through effector binding, adopts two interconverting states, a low-activity (T) state and a high-activity (R) state. Its activation through phosphorylation (inactive form of GPb to active GPa) is hormonally controlled. GP has several ligand-binding sites that can be exploited for inhibitor binding. These include the active site and sites for the physiological effectors such as AMP (allosteric site) and the glycogen binding site (storage site), as well as a hydrophobic site where purine-based compounds such as caffeine bind (inhibitor site).^10^

**Figure 1:**
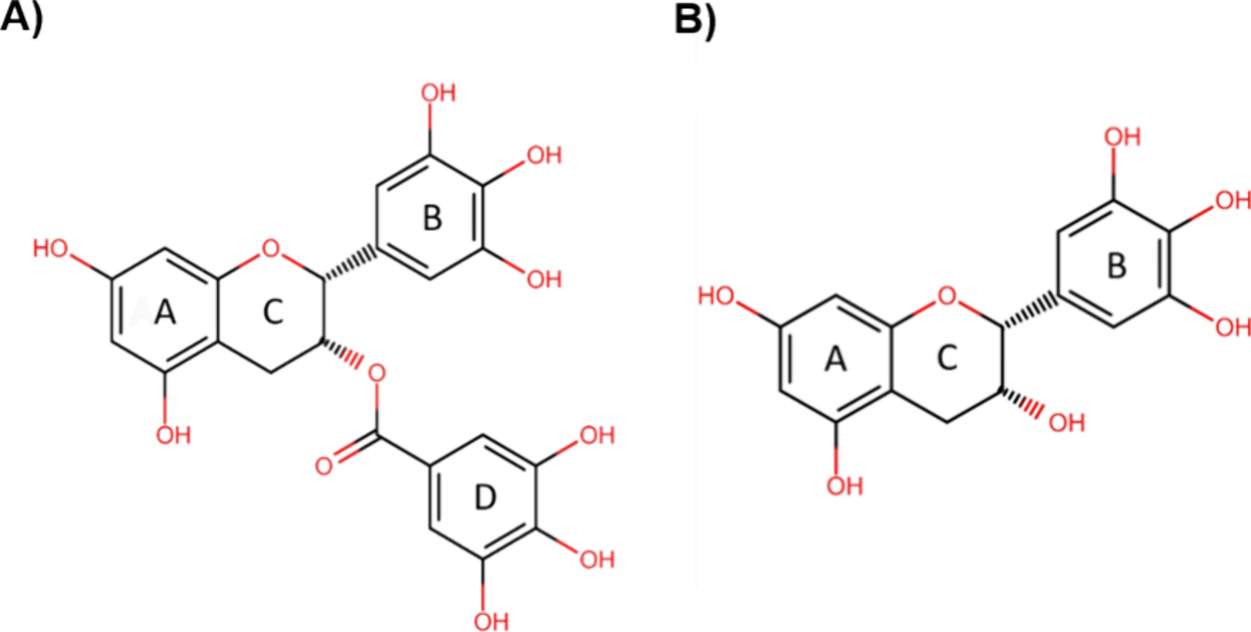
Chemical structures of flavonols: (A) EGCG [(−)-epigallocatechin gallate and (B) EGC [(−)-epigallocatechin]. The rings of each compound are presented by letters (A, B, C, and D).

Over the years, the remarkable “druggability” of GP has been revealed, as new binding sites have been identified by crystallographic studies. These include an indole-based compound binding site (new allosteric or drug site),^11^ a benzimidazole site,^12^ and, most recently, the quercetin binding site (QBS).^13^ Kinetic and crystallographic studies^14−18^ from our lab and others have revealed many potent inhibitors with *K*_i_ values in the order of nM toward GP. A number of these are natural compounds exhibiting a repertoire of different chemical scaffolds, including flavonoids, triterpenes, anthocyanins, and benzoic acid derivatives.^19,20^ The majority of the discovered GP inhibitors bind at the catalytic site that has a similar architecture in all isoforms; hence, the discovery of selective inhibitors that target only the human liver GP and not the muscle one remains a major challenge. Dietary flavanols are associated with a plethora of beneficial health effects. Epigallocatechin gallate (EGCG) and epigallocatechin (EGC) are the major bioactive flavanols of green tea (*Camellia sinensis*) with antioxidant, chemopreventive, anti-inflammatory, and antidiabetic effects.^21^ The structure of EGCG consists of four rings resulting from the esterification of EGC with gallic acid: the A and C rings constitute the benzopyran ring with a pyrogallol moiety at position 2, the B ring, with a gallate moiety at position 3, and the D ring (Figure 1).^22^

The substituents at positions 3′, 4′, and 5′ of the B ring and 3′,4′, and 5′ of the D ring are hydroxyl groups. In an early study^23^ by Jakobs et al., EGCG was shown to potently inhibit GP (IC_50_ values for GPa and GPb of 7.7 and 33.9 μM, respectively). Even though catechins are nonplanar molecules, catechin-3-gallates inhibit GPa activity to an extent similar to quercetin and luteolin. Back then, their inhibitory effect was conjectured to occur by binding at the inhibitor site due to the structural similarity with flavopiridol, a synthetic flavonoid derivative already known^24^ to bind at the same site. In light of the later identification of a different binding site for quercetin, we hypothesized that EGCG may bind at the QBS, and in this work, we provide the experimental evidence to support this. Using a combination of docking, kinetics, and crystallographic studies, we have examined the binding modes of the flavanols EGCG and EGC to GP isoforms, while the anticancer potential of EGCG was further assessed against GBM through studies with U87-MG cells.

## Materials and Methods

### Protein Purification

Rabbit skeletal muscle glycogen phosphorylase b (rmGPb) was isolated and then purified using the modified Fisher and Krebs method, as described.^25^ Human liver GP (hlGP) was expressed in *E. coli* and purified according to the protocol.^14^ The phosphorylated forms rmGPa and hlGPa were produced by phosphorylation using the constitutively active kinase domain of glycogen phosphorylase kinase (PhKγtrnc), as described.^14,26^

### Enzyme Assays

#### In Vitro GP Inhibition

The activities of rmGPb, rmGPa, and hlGPa were measured in the direction of glycogen synthesis by monitoring the release of inorganic phosphate, as described.^15^ rmGPb (3 μg/mL), rmGPa (3 μg/mL), and hlGPa (1 μg/mL) were assayed in the presence of glycogen (0.2% w/v) and AMP (1 mM). The reaction buffer contained 30 mM imidazole/HCl buffer (pH 6.8), 60 mM KCl, 0.6 mM EDTA, and 0.6 mM DTT. EGCG [(−)-epigallocatechin gallate] and EGC [(−)-epigallocatechin] were purchased by Extrasynthese (989-51-5/0981S and 970-74-1/0979S, respectively). In order to calculate the inhibition constant (*K*_i_) of EGCG with respect to GP, various concentrations of the inhibitor (0, 20, 40, 60, 80, 100 μM with respect to rmGPb and 0, 20, 40, 60, 80, 100 μM with respect to rmGPa) were assayed in various glucose-1-phosphate (G1P) concentrations (2, 3, 4, 6, 10 mM). Also, for the determination of the *K*_i_ of EGCG with respect to hlGPa, the inhibitor concentrations were 0, 20, 40, 60, and 80 μM at various G1P concentrations (1, 2, 3, 4, and 6 mM). The inhibitory effect of EGCG in synergy with well-established^27^ GP ligands was assessed through multiple inhibition studies. The activity of rmGPb was assayed in the presence of several concentrations of EGCG (0–100 μΜ) and varying concentrations of (a) caffeine (0–0.7 mM), (b) glucose (0–12 mM), and (c) AMP (0–0.5 mM). The inhibition constant (*K*_i_) of EGC with respect to rmGPb was calculated at EGC concentrations (0, 40, 80, 180, 250, 350 μΜ) and various G1P concentrations (2, 3, 4, 6, and 10 mM).

#### Inhibition of GP Activity in HepG2 Cells

HepG2 cells (1.5 million/dish) were grown in Dulbecco’s modified Eagle’s medium (DMEM), which was enriched with 25 mM glucose, 2 mM l-glutamine, 10% fetal bovine serum (FBS), and a penicillin/streptomycin antibiotic mixture. The cells were cultured at 37 °C in a 5% CO_2_ incubator. Sixteen hours after seeding, the medium was replaced by a high-glucose DMEM supplemented with 10 mM dexamethasone and 100 nM insulin that lacked l-glutamine and FBS in order to trigger the glycogen synthesis pathway. The cells were incubated overnight in high-glucose medium and after they were incubated in the presence or absence of different concentrations of EGCG (60 and 100 μM) and caffeine (200 μΜ) for 3 h in a glucose-free DMEM without phenol red, FBS, and l-glutamine, with the addition of glucagon (100 nM), to activate glycogenolysis. The cells were lysed and sonicated (4 cycles x 1s, 35% amplitude) and centrifuged at 15,600 rcf for 15 min at 4 °C. The activity of GPa in cell lysates (supernatant) was measured by glycogen synthesis, as described.^25^ The program GraFit^28^ was used for kinetic data statistical analysis. The cell cytotoxicity during the incubation with EGCG was estimated using the 3-[4,5-dimethylthiazole-2-yl]-2,5-diphenyltetrazolium bromide (MTT) assay.^29^

### Glioblastoma Cellular Studies

#### Cell Viability

Cell viability experiments were performed on the U87-MG GBM cell line. The cells were cultured in Eagle's minimum essential media (EMEM) with supplements and incubated at 5% CO_2_ at 37 °C. After a substantial growth period, the U87MG cells were seeded at a density of 5000 cells/well in a 96-well plate and incubated for 24 h. Media without cells were used as a blank, and cells and media without the treatment were used as a control. The cells were treated with EGCG diluted in media and incubated to establish a dose- and time-dependent toxicity effect at concentration ranges of 11, 22, 33, 44, 50, and 200 μM. Following 24 and 48 h of incubation with treatment, a 10 μL Presto Blue was added to the plates and incubated for 1 h, and then fluorescence response for Presto Blue was detected at *l*_Ex_ 535 nm and *l*_Em_ 612 nm. The % cell viability was calculated using eq 1 below.
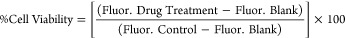
1

Mean cell viability versus drug concentration was plotted using Prism5 software (GraphPad Software, USA). IC_50_ values were calculated as the concentration at which 50% inhibition (decrease in cell proliferation) was achieved.

#### Scratch Assay

U87-MG cells were seeded at 200,000/2 mL/well in 6-well plates. The cells and media with no treatment were used as a control. Once the cells reached confluency, a scratch was made using a sterile tip. The detached cells were washed with phosphate-buffered saline (PBS), and the cells were treated with the IC_50_ concentration (24 h) of EGCG. Images were taken over predetermined time points (0, 6, 24, 48, and 72 h) at 4× magnification.

#### Flow Cytometry

The U87-MG cells were seeded at 120,000/well in a 12-well plate. After a 24 h incubation period, the cells were treated with IC_50_ (24 h) of EGCG and incubated for 24 h. Cells and media with no treatment were used as a control. Following treatment, the cells were washed, trypsinized, and centrifuged at 179*g* for 5 min. The cell pellets were resuspended and fixed by adding ice-cold 70% ethanol (diluted with PBS). The fixed samples were centrifuged at 179*g* for 5 min, washed with ice-cold PBS, and then centrifuged again at 179*g* for 5 min. The fixed cells were resuspended with PBS and transferred to nontissue culture plates/V-shaped. Propidium iodide (500 μg/mL stock) was added to each well, followed by adding RNAase (10 mg/mL), and incubated for 2 h, followed by testing the samples using a flow cytometer. Guava software was used for data analysis to determine the DNA content by fluorescence intensity (488 nm).

#### Statistical Analysis

For statistical analysis, SPSS was used with an independent sample *t-*test for cell cycle to identify significant differences between experimental groups: a two-way ANOVA and a Bonferroni posthoc test were used for cell viability. For the latter, significance between the time points, as well as treatment concentrations, was determined.

### X-ray Crystallography

rmGPb enzyme crystals were grown using the batch method in a 10 mM BES (pH 6.7) buffer, as described.^30^ The crystals were soaked in the crystallization medium enriched with (a) 5 mM EGCG, (b) 5 mM EGCG and 50 mM glucose, and (c) 5 mM EGCG and 10 mM caffeine and incubated for 2 days at 16 °C prior to their flash freezing in a nitrogen stream at 100 K nitrogen using 30% (v/v) DMSO used as a cryoprotectant. X-ray diffraction data were collected by using synchrotron radiation on the P13 beamline at PETRA III (EMBL Hamburg Outstation). The crystallographic suite CCP4^31^ was used for data analysis. Data integration and reduction were performed by the programs XDS^32^ and AIMLESS.^31^ The refinement of the structures was based on previously determined structure [Protein Data Bank (PDB) entry 7P7D]. The model building of the electron density map was performed using the program COOT.^33^ Ligand molecule coordinates and topologies were created with the program ACEDRG of the CCP4 suite and were fitted to the electron density maps. The crystallographic refinement of the complexes was performed using maximum likelihood methods by REFMAC5.^34^ The programs CONTACT, also in the CCP4i software suite, and PLIP^35^ were used for the determination and calculation of protein–ligand interactions. The final structures were optimized using the PDB-REDO server.^36^ The program PyMOL^37^ was used in order to create graphic designs of the structure.

### *In Silico* Studies

#### Protein Preparation

Muscle and liver isoforms of GP were prepared for computations using Schrödinger’s Protein Preparation Wizard^38^ applied to the newly solved cocrystallized complex with EGCG (rmGPb, PDB entry: 8QMU, 2.0 Å resolution) and the previously reported complexes with quercetin (rmGPb, PDB entry: 4MRA, 2.34 Å resolution) and *N*-acetyl-β-d-glucopyranosylamine (hlGP, PDB entry: 1FC0, 2.40 Å resolution). Water molecules within 5 Å of the native ligands were initially retained (deleted in docking calculations, unless specified), bond orders were assigned, and hydrogens were added, with protonation states for basic/acidic residues assigned from calculated PROPKA^39^ p*K*_a_ values at a pH of 7. Subsequent optimization of hydroxyl groups, His residue protonation states, and potential for its side-chain C/N atom flips, as well as possible side-chain O/N atom flips in Asn and Gln residues, was based on optimizing hydrogen-bonding patterns. Finally, an Impref minimization of the prepared complexes was performed using the OPLS3e force field^40^ to relax any steric clashes and/or bad contacts. At the end of the minimization, the RMSDs (heavy atoms) were within 0.3 Å of the crystallographic positions.

#### Ligand Preparation

All ligands were prepared for docking using Maestro and Macromodel 13.0.^38^ Each ligand was minimized using 200 steps of the truncated Newton conjugate gradient (TNCG) method,^41^ with the OPLS3e force field and water solvation effects accounted for using the generalized Born/surface area (GB/SA) model.

#### Glide Docking Calculations

Docking calculations were performed using Glide 8.9.^38^ Using the prepared GPb proteins from PDB entries: 8QMU, 4MRA, and 1FC0 (as described above), the shape and properties of the QBS were mapped onto a grid with dimensions of ∼23.5 × 23.5 × 23.5 Å that were centered on the native ligands; in the case of 1FC0 with no ligand bound at QBS, the position of EGCG from the solved rmGPb–EGCG complex (PDB entry: 8QMU) was superimposed into the binding site for this purpose. Standard parameters were used that included default atomic charges and van der Waals scaling (0.8) for nonpolar ligand atoms to include modest induced-fit effects. Calculations were performed in SP mode with postdocking minimization. Up to five output poses per ligand were saved in each docking calculation.

#### Induced-Fit Docking Calculations

Induced-fit docking (IFD) calculations of EGCG to GP from the solved rmGPb–quercetin complex (PDB code: 4MRA) were performed.^38^ The IFD calculations consisted of three stages. In stage I, an initial Glide 8.9 SP docking was performed with a maximum of 20 poses saved. The Arg551 residue was selected for mutation to Ala during this initial docking (rebuilt in stage II). In stage II, the binding site residues Arg551 and Glu120 were refined using Prime 6.2.^42^ Finally, in stage III, up to 10 structures from stage II within 30 kcal/mol of the lowest energy structure were employed in Glide-SP ligand redocking calculations. The final predicted complexes ranked by IFD scores were then analyzed in terms of structure and protein–ligand binding interactions.

## Results and Discussion

### Kinetic Analysis and Synergism

**Figure 2:**
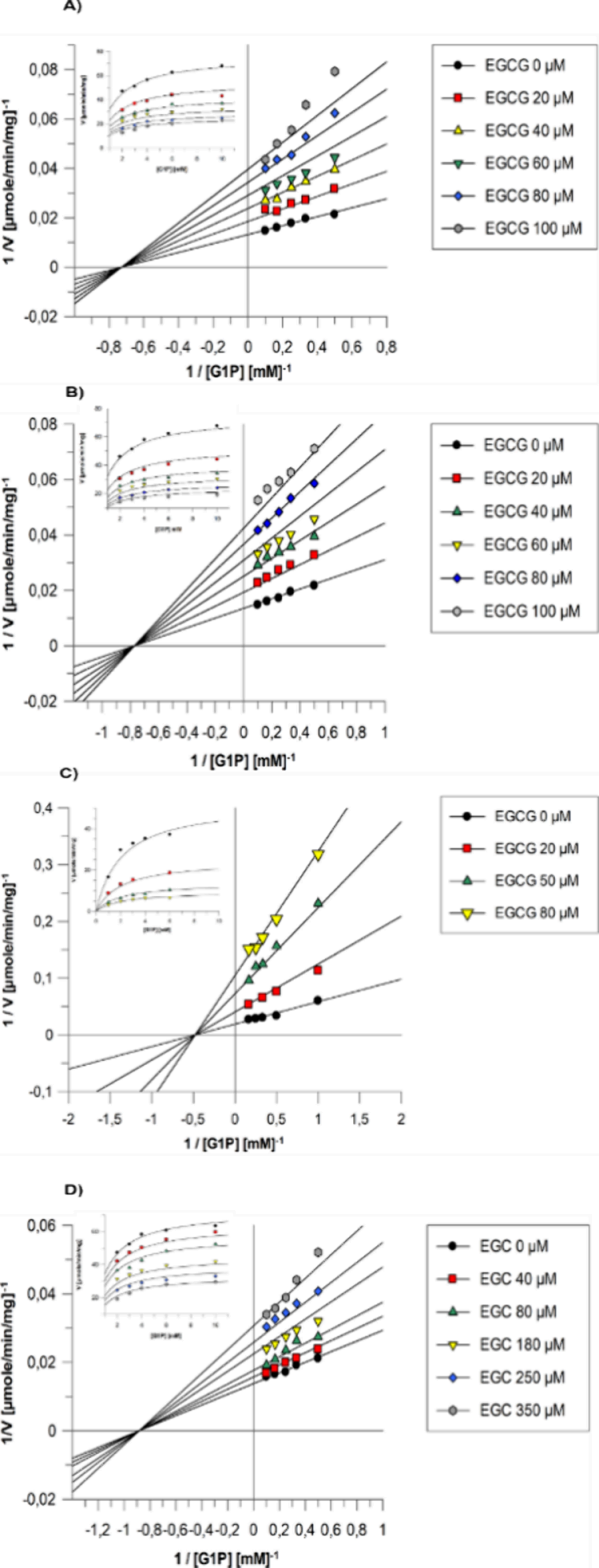
Inhibition of (A) rmGPa, (B) rmGPb, and (C) hlGPa by EGCG and (D) by EGC. Lineweaver–Burk plots of reciprocal velocities versus reciprocal G1P concentrations at different EGCG concentrations and constant concentrations of glycogen (0.2% w/v) and AMP (1 mM) for rmGPb. (Α, Β) EGCG concentrations were 0 μM, 20 μM, 40 μM, 60 mM, 80 μM and 100 μΜ. (C) EGCG concentrations were 0, 20, 50 and 80 μM. (D) EGC concentrations were 0, 40, 80, 180, 250, and 350 μΜ. The diagrams of each velocity (*V*) in *y*’*y* at different substrate concentrations in *x*’*x* are shown in the insets.

Κinetic experiments with rmGPb and rmGPa showed that EGCG potently inhibited GP *K*_i_ = 49.8 ± 1.6 μM for rmGPb and *K*_i_ = 46.7 ± 1.4 μΜ for rmGPa, hence having similar affinity for both muscle GP forms (Table 1). In comparison, the *K*_i_ value of EGC was calculated to be 285.5 ± 14.1 μΜ for rmGPb and the IC_50_ value for hlGPa was 193.8 ± 3.5 μM, revealing that the gallate group of EGCG confers potency. Furthermore, the inspection of the Lineweaver–Burk plots (Figure 2) revealed that EGCG and EGC are noncompetitive inhibitors.

**Table 1:** Values of Inhibition Constants (*K*_i_) for EGCG, EGC, and Quercetin toward GP Isoforms

GP isoform	EGCG, *K*_i_ (μΜ)	EGC, *K*_i_ (μΜ)	Quercetin, *K*_i_ (μΜ)^44^
rmGPa	46.7 ± 1.4		32.93 ± 2.49
rmGPb	49.8 ± 1.6	285.5 ± 14.1	69.12 ± 4.71
hlGPa	17.6 ± 0.7		43.52 ± 1.65

**Figure 3:**
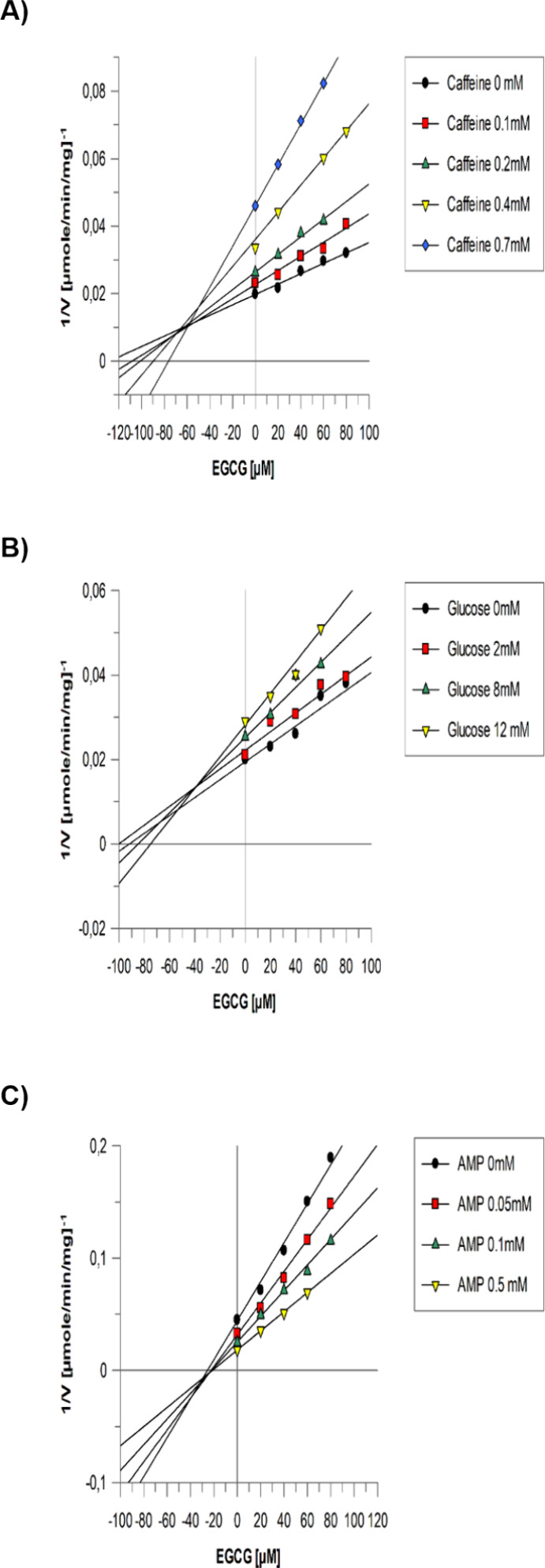
Multiple inhibition studies. Dixon plots of reciprocal velocity versus EGCG concentrations at different concentrations of (A) caffeine, (B) glucose, and (C) AMP. In panels (A, B), the samples were assayed at constant concentrations of Glc-1-P (10 mM), AMP (1 mM), and glycogen (0.2% w/v). (C) Concentrations of AMP varied from 0 to 0.5 mM.

The inhibitory properties for more potent EGCG were further investigated with respect to well-known inhibitors glucose, caffeine, and AMP that bind at the catalytic, inhibitor, and allosteric GP sites, respectively. The Dixon plots of 1/*v* versus [EGCG] at varying concentrations of caffeine (Figure 3a) showed that the curves intersect above the horizontal axis, revealing cooperative inhibitor binding. The Dixon plots (Figure 3b) in the case of glucose are indicative of synergistic inhibition of a competitive inhibitor (glucose) with a noncompetitive one (EGCG). The Dixon plots of 1/*v* versus [EGCG] at varying AMP concentrations (Figure 3c) showed curve intersections below the horizontal axis, indicating that binding of an inhibitor hinders the binding of the other.^43^

**Figure 4:**
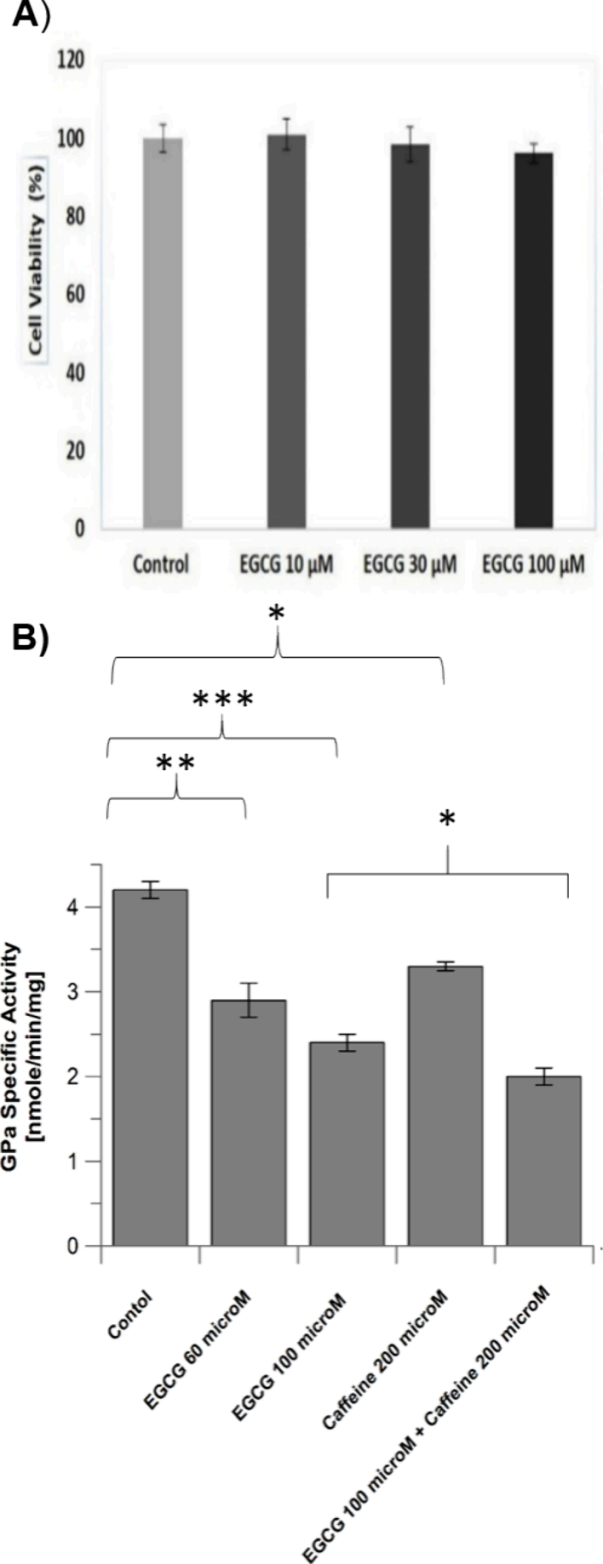
(A) Viability of HepG2 cells upon 3 h treatment with EGCG measured by applying the MTT assay. The vehicle-treated control cells were set at 100%. No statistical differences were observed. (B) Diagram of hlGPa activity in the presence of increasing concentrations of EGCG, caffeine, and simultaneous incubation of HepG2 cells with EGCG and caffeine. GPa activity is expressed as nmol/min/mg protein and calculated using 5 time points and errors (shown on bars). The same experimental conditions were conducted twice for each sample. Data were analyzed by one- and two-way ANOVA, followed by Tukeys’s posthoc test, using StatPlusLE 7.3.0 software, and expressed as mean ± SD (*n* = 2). *p ≤ 0.05,** *p* ≤ 0.01; *** *p* ≤ 0.001.

Kinetic studies with the physiological target hlGPa isoform also revealed that EGCG is a noncompetitive inhibitor with *K*_i_ = 17.6 ± 0.7 μΜ (Table 1 and Figure 2c) about 3 times lower *K*_i_ value compared with the muscle isoforms. This difference in *K*_i_ values among the liver and muscle GP isoforms is more pronounced in comparison to quercetin,^44^ which is almost equipotent for rmGP and hlGPa. This new result is encouraging, as it indicates an element of tissue selectivity for EGCG-like inhibitors. The efficacy of EGCG to inhibit GP was further assessed (Figure 4) in ex vivo system of hepatocarcinoma HepG2 cells in culture. Prior to experiments, the toxicity of EGCG toward HepG2 cells was assessed, and no toxicity was observed upon 3 h up to 100 μΜ. The activity of GP was assessed after treatment with 60 and 100 μM EGCG, as described in the Materials and Methods section, and the results showed 31.8 and 43.5% reduction in GP activity, respectively. Furthermore, the effect of EGCG on GP inhibition in HepG2 cells in the presence of caffeine was also assessed. It is demonstrated in Figure 4B that the treatment of cells with 200 μM caffeine caused 22.3% inhibition in GP activity, and the percentage of inhibition was increased to 52.9% after cotreatment with 100 μM EGCG. The results are in agreement with in vitro assays, showing that caffeine and EGCG are nonexclusive inhibitors that bind at different sites of GP, and their synergistic effect is also exhibited in HepG2 cells. To our knowledge, it is the first time that synergy in cells was shown for inhibitors other than glucose.

### Effect of EGCG on Glioblastoma

**Figure 5:**
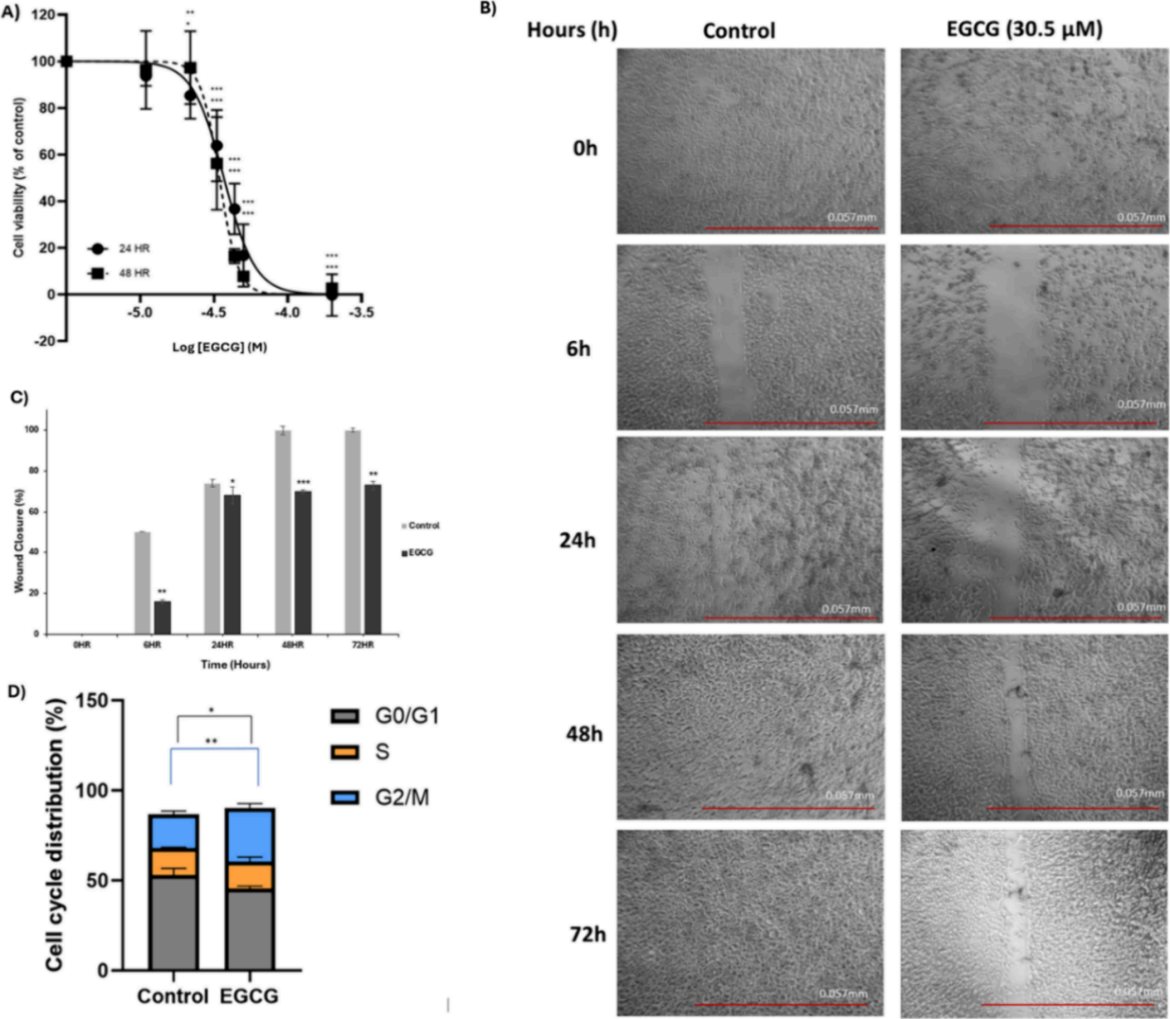
Effect of EGCG on GBM. (A) % Cell viability and effect of EGCG over 24 and 48 h against U87-MG cells (*N* = 3, mean/SD). The cells were treated with 11, 22, 33, 44, 50, and 200 μM concentrations. IC_50_ values of 30.5 μM ± 0.4 and 24 μM ± 2.7 at 24 and 48 h were observed, respectively. (B) Wound/scratch assay (*N* = 3) for U87-MG cells treated with the IC_50_ concentration (30.5 μM) of EGCG compared against a control over 72 hours. The images are captured at 4× magnification (scale 0–10 mm in 0.1 mm). (C) Bar chart representing the % wound closure following treatment with EGCG IC_50_ concentration (30.5 μM) over a period of 72 h in comparison with control nontreated U87-MG cell lines. (D) Cell cycle analysis of U87-MG cells (*N* = 3, mean/SD), represented as the percentage prevalence of G0/G1, S and G2/M cell cycle phases. EGCG-treated U87-MG cells were compared for significance against untreated control cells. *P*-values ≤0.05 were considered statistically significant with significance indicated in figures as ns, *p* > 0.05, * *p* ≤ 0.05, ** *p* ≤ 0.01, and *** *p* ≤ 0.001.

The anticancer potential of EGCG against U87-MG cells was determined following 24 and 48 h of treatment. Each time point presented a significant reduction in overall cell viability following treatment with EGCG, with a significant reduction in the cell viability of the treated cells from log [EGCG] = −4.7 (i.e., 22 μM) onward (Figure 5A).

U87-MG cells demonstrated a significant decrease in cell viability when incubated with EGCG with IC_50_ values of 30.5 ± 0.4 and 24 ± 2.7 μΜ at 24 and 48 h, respectively. These results were in line with studies that reported a decrease in cell viability at 50 μΜ after a 24 h period^45^ and at 25 μM after 48 h of treatment.^46^ It is worth mentioning that there was no significant difference (*p* > 0.05) between 24 and 48 h IC_50_ values, indicating that the inhibition in the cell viability was achieved in the first 24 h, following treatment with EGCG, and slightly increased following 48 h. From the wound/scratch assay experiments (Figure 5B,C), the inhibition of cell migration via EGCG of U87-MG cells was tested over various time points. Following the treatment with IC_50_ concentration of EGCG (30.5 μM) at an early time point (6 h), the U87-MG cells showed a significant reduction in cell migration, where the % wound closure was 16% (*p* < 0.005), as demonstrated in Figure 5C, in comparison to the control-non-treated U87-MG cell lines, where the % wound closure was around 50%. In addition, at 24 h of treatment, a 68% wound closure (*p* < 0.05) was observed; a similar 67.5% wound closure at 24 h has been reported for another natural product GP inhibitor luteolin (IC_50_(GPa) = 15.6 μM; IC_50_(GPb) = 28.8 μM)^23^ in the same cell line at 30 μM concentration.^47^ Interestingly, the control cells exhibited 100% wound closure (no visible wound) in both 48 and 72 h (Figure 5B,C), while U87-MG cells treated with EGCG still exhibited a significant reduction of % wound closure with 70 and 73% (*p* < 0.001 and *p* < 0.005) for 48 and 72 h, respectively. This suggests the potential of EGCG to limit cell proliferation and migration in GBM. During the cell cycle study (Figure 5D), the control U87-MG had the highest percentage of cells in the G0/G1 phase at 53.067 ± 3.623%, followed by 18.747 ± 1.786 and 14.960 ± 0.442% for G2/M and S phases, respectively. In comparison, U87-MG cells treated with IC_50_ of EGCG showed significant differences, with a cell prevalence of 45.770 ± 1.683% for G0/G1 (predominant phase), followed by 29.810 ± 3.352% at the G2/M phase and 14.885 ± 3.472% for the S phase. EGCG-treated cells were therefore associated with a decrease of cells in the G0/G1 phase and concomitant increases in the G2/M phase. In line with the potential role of GP, EGCG is known to increase the accumulation of p53 in cancerous cell lines.^48^ In GBM in vitro and in vivo studies, liver isoform depletion led to increased levels of p53; in vivo, consequent glycogen accumulation led to increased reactive oxygen species that contributed to a p53-dependent induction of senescence and impaired tumorigenesis.^6^ Nevertheless, the anticancer therapeutic potential of GP inhibition should be further explored in the future through the discovery of less promiscuous than EGCG and more specific GP inhibitors.

### Binding at the Quercetin Binding Site of rmGPb

In order to elucidate the structural basis of inhibition of GP by EGCG, we determined, using X-ray crystallography, the structure of the rmGPb-EGCG complex (PDB entry: 8QMU) at a 2.0 Å resolution and the complexes rmGPb-EGCG-glucose (PDB entry: 8R53) and rmGPb-EGCG-caffeine (PDB entry: 8R6V) at 2.0 and 2.5 Å, respectively. The data processing and refinement statistics are shown in Table S1. A–C. Inspection of the 2Fo–Fc electron density maps in all complexes revealed a positive peak at the site of rmGPb, as previously recognized^13^ to bind quercetin. The QBS is located close to the protein surface about 15 Å from the active site, 43 Å from the allosteric site, and 32 Å from the inhibitor site. It forms a groove constellated by residues Lys544, Arg551, Lys655, and Tyr548 on one side and by Glu120 and Glu123 on the other side. Upon binding, EGCG makes a total of 107 van der Waals (vdw) interactions (14 polar–polar, 39 nonpolar–nonpolar, and 54 polar–non polar interactions). A detailed description of protein–ligand interactions is given in Table S2 in the Supporting Information. The ring B hydroxyl oxygen atoms (O1), (O7), and (O37) and the hydroxyl oxygen (O44) of the gallate group form 5 hydrogen bonds (hb) with protein atoms (Table S2.A–Supporting Information).

**Figure 6:**
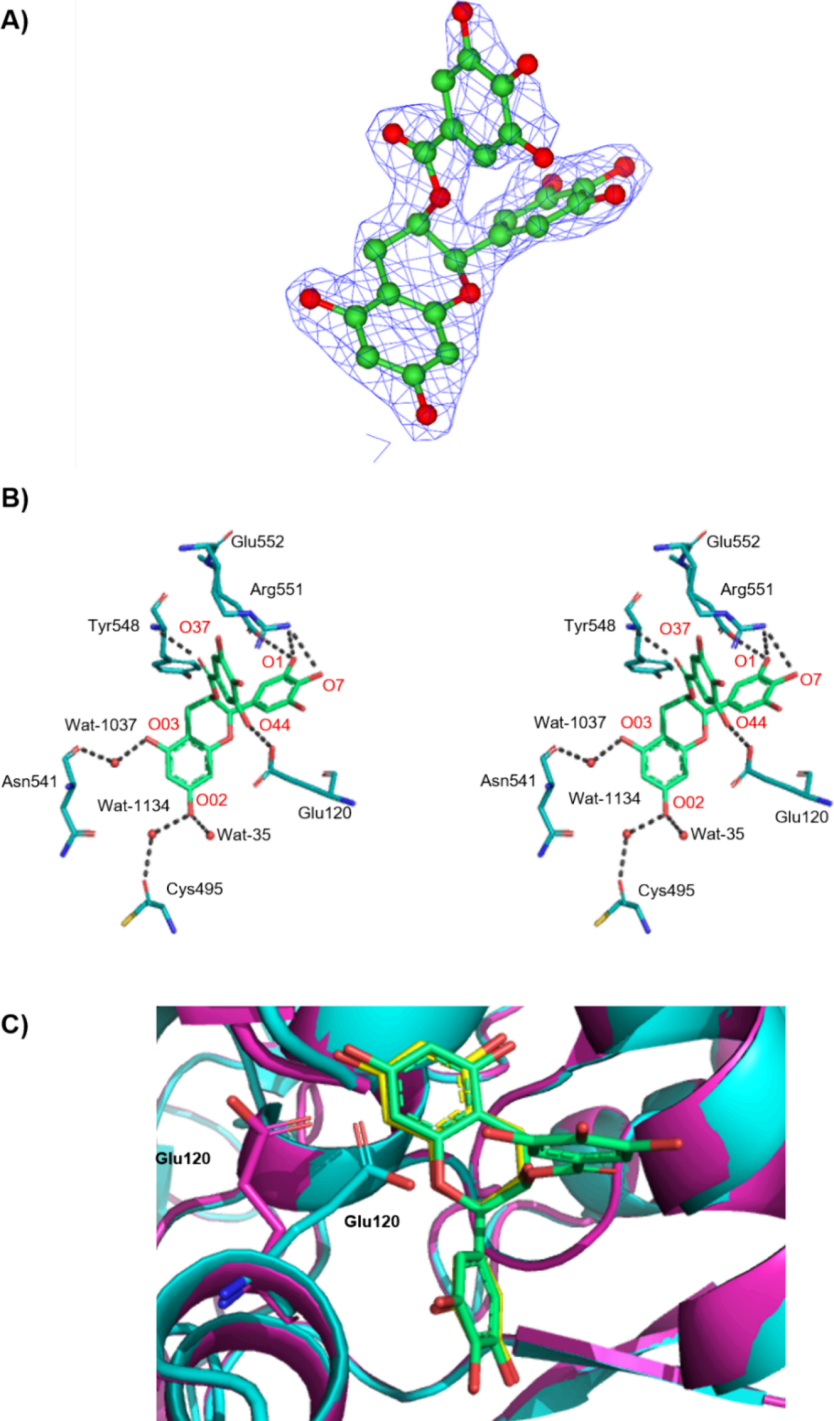
Binding of EGCG at the QBS of rmGPb. (A) Fo-Fc electron density map, contoured at 3σ, for the bound state of EGCG (green) at rmGPb. (B) Stereo diagram of binding of EGCG to the QBS of rmGPb. Hydrogen-bonding interactions are represented by dotted lines and water molecules as spheres. (C) Superposition of EGCG-rmGPb (cyan) and EGC-rmGPb (magenta) complexes, where a shift of Glu120 was observed due to the gallate ring of EGCG.

In addition, 2 water-mediated bridges with protein residues are formed, namely, O02-Wat1037-Cys495 (O) and O03-Wat1134-Asn541 (OD1). O02 also forms an hb with Wat35. Overall, 8 hb are formed. Aromatic rings A and B are positioned in 4.06 and 3.20 Å of positively charged Lys544 and Arg551, respectively, forming cation−π interactions. (Figure 6B).

Superposition of the native rmGPb T state (PDB entry 7P7D) structure onto the rmGPb-EGCG complex structure, residues (7-836), showed RMSD values of 0.244 Å on Cα atoms and 0.260 and 0.613 Å for main-chain and side-chain atoms, respectively. Structural determination of the complexes of rmGPb-EGCG-glucose and rmGPb-EGCG-caffeine demonstrated that EGCG can bind at the QBS simultaneously with glucose at the catalytic site or caffeine at the inhibitor site, in good agreement with the synergistic behavior from the multiple inhibition studies described above. The ligand–protein interactions for all solved complexes are summarized in Tables S2 and S4 Supporting Information. Superposition of the complex of rmGPb-EGCG with the rmGPb-EGCG-glucose complex showed no significant overall conformational changes with RMSD values of 0.205 Å on Cα atoms and 0.235 and 0.598 Å for main-chain and side-chain atoms, respectively. Superposition of rmGPb-EGCG-glucose with rmGPb-glucose (PDB entry 2PYD) showed no differences in protein–glucose interactions, while the RMSD values for Cα atoms were 0.174, 0.225, and 0.963 Å for main-chain and side-chain atoms, respectively. Likewise, the superposition of the binary complex of rmGPb-EGCG with the rmGPb-EGCG-caffeine complex showed no significant overall conformational changes with RMSD values of 0.234 Å on Cα atoms and 0.268 and 0.692 Å for main-chain and side-chain atoms, respectively. No significant differences in the number of vdw interactions and no changes in hb interactions with protein atoms were observed although the water-mediated hb interactions were less due to a lower resolution for the rmGPb-EGCG-caffeine complex. In addition, the superposition of rmGPb-EGCG-caffeine with rmGPb-caffeine (PDB, 1GFZ) showed RMSD values of 0.406 Å on Cα atoms and 0.427 and 1.217 Å for main-chain and side-chain atoms, respectively. Interestingly, in the case of the rmGPb-EGCG-caffeine complex, caffeine forms an extra water-mediated hb and more (23) vdw interactions at the inhibitor site when EGCG is bound. This difference can be attributed to the effect of the EGCG binding on increasing the caffeine interactions with the protein in favor of T-state stabilization although differences in experimental conditions for both complexes should also be considered.

**Figure 7:**
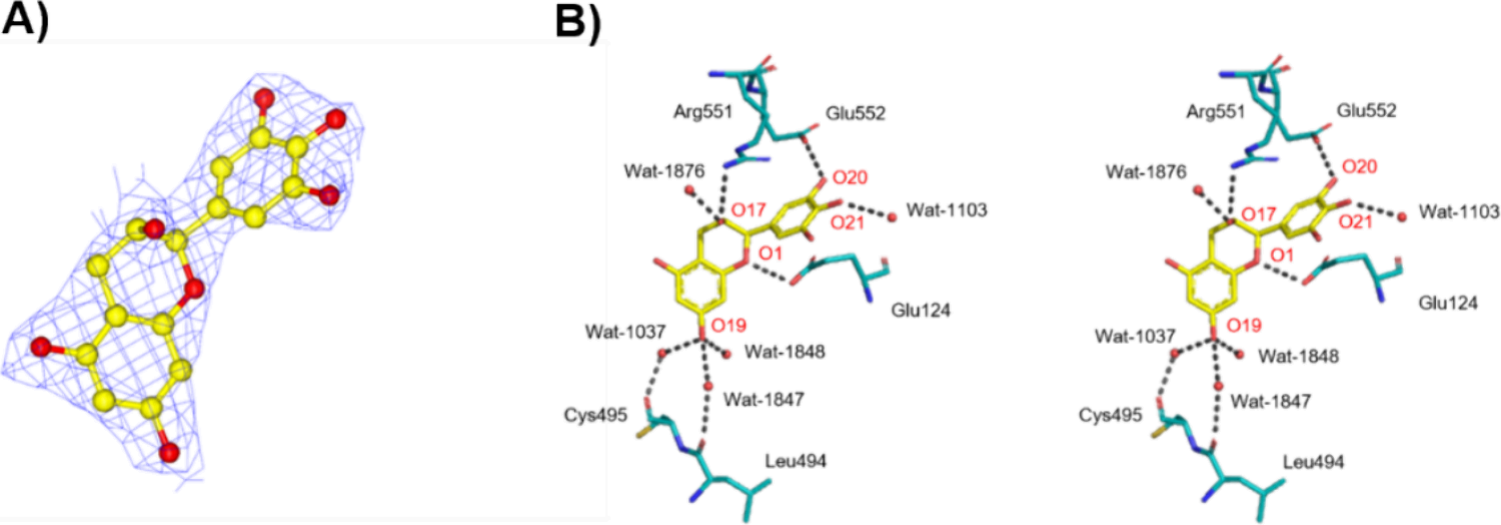
Binding of EGC at the QBS of rmGPb. (A) Fo-Fc electron density map, contoured at 3σ, for the bound of EGC (yellow) at rmGPb. (B) Stereo diagram of the binding of EGC to the QBS of rmGPb. Hydrogen-bonding interactions are represented by dotted lines and water molecules as spheres.

The structure of the rmGPb-EGC complex (PDB entry: 8R52) was also determined with a 2.1 Å resolution (Table S1.D, Supporting Information). The 2Fo–Fc map also showed that EGC binds at the QBS although a high concentration of EGC (15 mM, 3× more than EGCG) was needed in order to achieve binding, reflecting its low potency toward GP. Superposition of the rmGPb-EGC complex structure with the rmGPb T state (PDB entry 7P7D) and residues (7-836) showed RMSD values of 0.229 Å on Cα atoms and 0.249 and 0.665 Å for main-chain and side-chain atoms, respectively. EGC forms 22 less van der Waals interactions (overall 85, 7 polar–polar, 37 nonpolar–nonpolar, and 41 polar–nonpolar) compared to EGCG. The lack of the gallate group and its hydroxyls explains differences mostly in polar interactions and also in hb bonding (Table S2.B, Supporting Information). EGC forms 3 hb with protein residues in comparison to 5 of EGCG. The hydroxyl oxygen (O17) forms a hb with Wat1876. The hydroxyl oxygen O19 of ring A makes 2 water-bridged interactions: the water-bridged interactions O19-Wat1037-Cys495 (O) and the water-bridged interactions O19-Wat1847-Leu494 (O) and the hydrogen-bridged interactions 1 hb with Wat1848. In addition, (O21) forms 1 hb with Wat1103, and the overall EGC forms 8 hb interactions. Arg551 is at a 3.58 Å distance from ring B and Lys544 is at a distance of 3.23 Å from ring A, participating in π–cation interactions (Figure 7).

### Contribution of the Gallate Group in EGCG to Binding

The gallate group (ring D) of EGCG forms 2 extra hydrogen bonds through its hydroxyl oxygens (O37) and (O44) compared with the nongallated EGC, with residues Tyr548 (N) and Glu120 (OE2), respectively. Superposition of the binary complexes of rmGPb-EGCG with rmGPb-EGC reveals a shift (RMSD, 3.31 Å) of the Glu120(OE2) side chain upon EGCG binding due to its hydrogen-bonding interaction with hydroxyl oxygen (O44) of the gallate group (Figure 6C). The same shift (RMSD, 2.77 Å) of Glu120 is also observed in comparison with the rmGPb-quercetin complex. Glu120 is located near the entrance of QBS, and its participation in hb confers plasticity to the site that could be exploited upon ligand binding.

### Comparison with Quercetin Binding

Superpositions of rmGPb-EGCG and rmGPb-EGC with the rmGPb-quercetin complex (PDB entry 4MRA) showed that EGCG and EGC adopt the same binding orientation with no overall significant conformational changes. In comparison to quercetin, EGCG having an extra ring (ring D) forms overall more vdw interactions (107 compared to 63 for quercetin). Upon binding, quercetin exploits 6 hydrogen bonds (3 with water molecules) and π–cation interactions with Lys544, Lys655, and Arg551 although it does not form a hb with Arg551. EGCG forms 2 extra hb with protein residues exhibiting greater potency (×1.4 times).

### Differences in EGCG Binding for Muscle and Liver GP Isoforms

**Figure 8:**
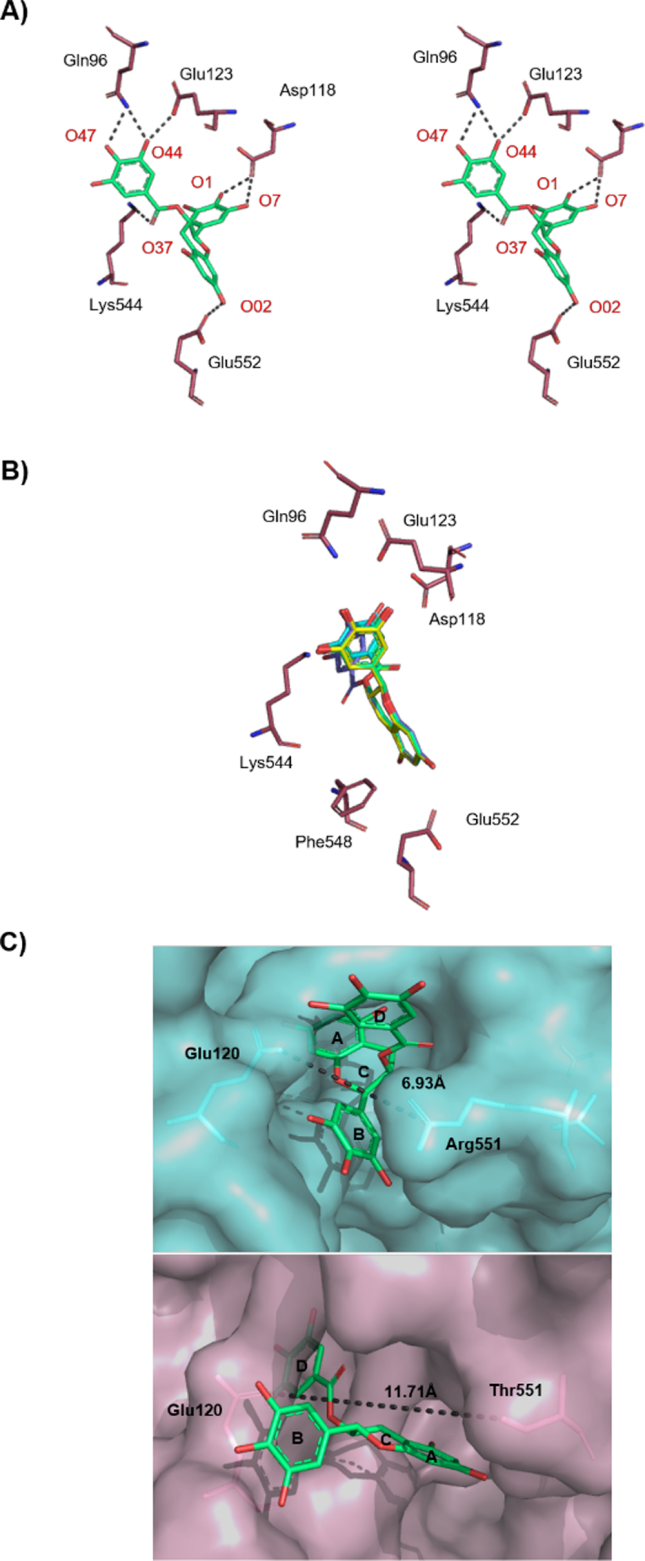
Different binding modes of EGCG for muscle and liver GP isoforms. (A) Stereo diagram of the top-ranked predicted binding pose of EGCG (green) binding to the QBS of hlGPa from Glide-SP docking. Hydrogen-bonding interactions are represented by dotted lines. (B) Superposition of the other four predicted poses for binding of EGCG to hlGPa. (C) Different binding modes of EGCG binding between rmGPb (cyan) and hlGPa (light pink) is demonstrated. The difference in the distance between Arg551 and Glu120 in the muscle isoform and between Thr551 and Glu120 in the liver isoform is represented by dotted lines.

Sequence alignment using T-Coffee^49^ of the rabbit/human muscle GP and human GP liver isoforms (Figure S1, Supporting Information) revealed that although most of the residues decorating the QBS are conserved, Tyr548 in muscle is a Phe in liver, but, most importantly, the key interacting Arg551 residue at the entrance of the site in muscle GP is in fact a Lys in the brain and a Thr in liver GP isoform. This shorter polar Thr “opens” the entrance of the QBS, allowing for different interactions with ligands. Actually, the distance between the gating residues Arg551 (CZ) and Glu120 (OE2) is 6.97 Å in muscle isoform and is 4.63 Å shorter compared to a distance of 11.71 Å between Thr551 (OG1) and Glu120 (OE1) in liver isoform. This observation leads to the assumption of a different binding mode of EGCG for muscle and liver isoforms, justifying differences in *K*_i_ values (Table 1), as shown by kinetic analysis. To probe the potential for EGCG binding differences to the muscle and liver GP isoforms, docking calculations of EGCG to the solved human liver GP isoform of PDB entry: 1FC0 were performed. As an initial test of the application of the Glide-SP docking method for this application, EGCG was redocked to the solved muscle EGCG-rmGPb complex reported here (PDB entry: 8QMU) and reproduced well the crystallographic conformation, with a ligand RMSD (heavy atoms) of 0.683 Å for the top-ranked binding pose. As a more challenging test, EGCG was cross-docked to the solved muscle GPb structure from its solved complex with quercetin (PDB entry: 4MRA). In this case, none of the predicted poses reproduced the EGCG crystallographic conformation. It was speculated that the positioning of Arg551 was a key factor in this regard so that IFD calculations were performed toward confirming this hypothesis. In the IFD, Arg551 was mutated to an Ala residue in the initial Glide-SP docking stage before the residue was rebuilt, and the complex was refined using Prime before a final Glide-SP docking stage (c.f*.* computational details). The Glu120 residue due to its shifted position (Figure 6C) was also included in the refinement. Applying this protocol, ten predicted protein–ligand complexes were returned, one of which was the crystallographic pose (PBD entry: 8QMU) with a ligand RMSD (heavy atoms) of 0.961 Å (after protein backbone superimposition). Crucially, Arg551 side-chain orientations were diverse in the returned predicted complexes, and only when Arg551 was close to its correct crystallographic position was the correct pose of EGCG predicted, highlighting the key role of Arg551 on ligand binding modes. Arg551 is Thr551 in the liver GP isoform, and hence, based on the above analysis, we expected that a different ligand binding pose of EGCG would be predicted for docking to human liver GP. This indeed was the case. Five ligand poses were saved, all of which were close to the same location and binding shape in the QBS but different from those observed for muscle GP. Inspection of the binding poses revealed that EGCG adopted a different binding orientation and exploited a different network of protein–ligand interactions compared to rmGP (Figure 8). The top-ranked binding pose of EGCG exploits 7 hb interactions with protein atoms (Table S3, Supporting Information).

This binding orientation allows 2 more direct hydrogen bonds with protein atoms compared to those in the rmGPb complex (the rest are water-mediated). Lys655 (NZ) is positioned at 4.33 Å from the center of ring D forming a cation−π interaction, further stabilizing the complex (Figure 8A). The nearby Phe548 can also be exploited for π–stacking interactions (T-shaped), as revealed by docking poses 2–5 (Figure 8B). Hence, the differences in *K*_i_ values for muscle versus liver GP isoforms (selectivity of EGCG for liver isoform) seem to be well explained by structural analysis, and in particular, the influence of Thr551 versus Arg551 in ligand binding at the QBS (Figure 8C). Hence, the development of EGCG-like inhibitors that target QBS could be considered a good starting point for the design of liver-selective GP inhibitors with the potential against different diseases. Overall, EGCG binding at the QBS offers new opportunities for the design of liver-selective GP inhibitors while also revealing a potential role of ligand-induced inhibition of GP against cancers such as GBM.

## Supporting Information Available

Sequence alignment figure of GP isoforms (Figure S1); summary of the diffraction, data processing, and refinement statistics for the rmGPb complexes (Table S1. A–D); all hydrogen bonds that EGCG form with rmGPb residues (Table S2); all hydrogen bonds that EGCG form with hlGPa residues (Table S3); and all the interactions that EGCG, EGC, glucose, and caffeine form with glycogen phosphorylase residues in the crystal (Table S4. A.–I.); locations of different binding sites n GP monomer (Figure S2); further figures comparing EGCG and quercetin binding at QBS (Figures S3-S4) (PDF)

## Abbreviations used

DMEM: Dulbecco’s modified Eagle’s medium
EGC: epigallocatechin
EGCG: epigallocatechin gallate
EMEM: Eagle's minimum essential media
FBS: fetal bovine serum
G1P: glucose-1-phospate
GB/SA: generalized born/surface area model
GBM: glioblastoma
GP: glycogen phosphorylase
hlGP: human liver GP
hlGPa: human liver glycogen phosphorylase GPa
MTT: 3-[4,5-dimethylthiazole-2-yl]-2,5-diphenyltetrazolium bromide
PBS: phosphate-buffered saline
PhKγtrnc: constitutively active kinase domain of glycogen phosphorylase kinase
PDB: Protein Data Bank
QBS: quercetin binding site
rmGPb: rabbit skeletal muscle glycogen phosphorylase b
TNCG: truncated Newton conjugate gradient method method
T2D: type 2 diabetes
IFD: induced-fit docking
