# Supplementary material for: Evidence for the Quercetin Binding Site of Glycogen Phosphorylase as a Target for Liver-Isoform-Selective Inhibitors against Glioblastoma: Investigation of Flavanols Epigallocatechin Gallate and Epigallocatechin
Source: J Agric Food Chem. 2024 Oct 21;72(43):24070–81. doi: 10.1021/acs.jafc.4c06920 (PMC11528470; doi:10.1021/acs.jafc.4c06920)
Supplement: Supplementary file 1 — jf4c06920_si_001.pdf [file jf4c06920_si_001.pdf]

Supporting Information

Evidence for the quercetin binding site of glycogen phosphorylase as a target for liver isoform selective inhibitors against glioblastoma: flavanols epigallocatechin gallate (EGCG) and epigallocatechin (EGC) investigated.

Serafeim Alexopoulos,[1] Megan McGawley,[2] Roshini Mathews,[2] Souzana Papakostopoulou[1], Symeon Koulas[1], Demetres D. Leonidas[1], Tamara Zwain,[2] Joseph M. Hayes[2] and Vasiliki Skamnaki.[1*]

[1]*Department of Biochemistry and Biotechnology, University of Thessaly, Biopolis, 41500, Larisa, Greece.*

[2] *School of Pharmacy & Biomedical Sciences, University of Central Lancashire, PR1 2HE, Preston, UK.*

*Email: vskamnaki@bio.uth.gr

Figure S1, S2, S3, S4. Table S1, S2, S3 and S4.

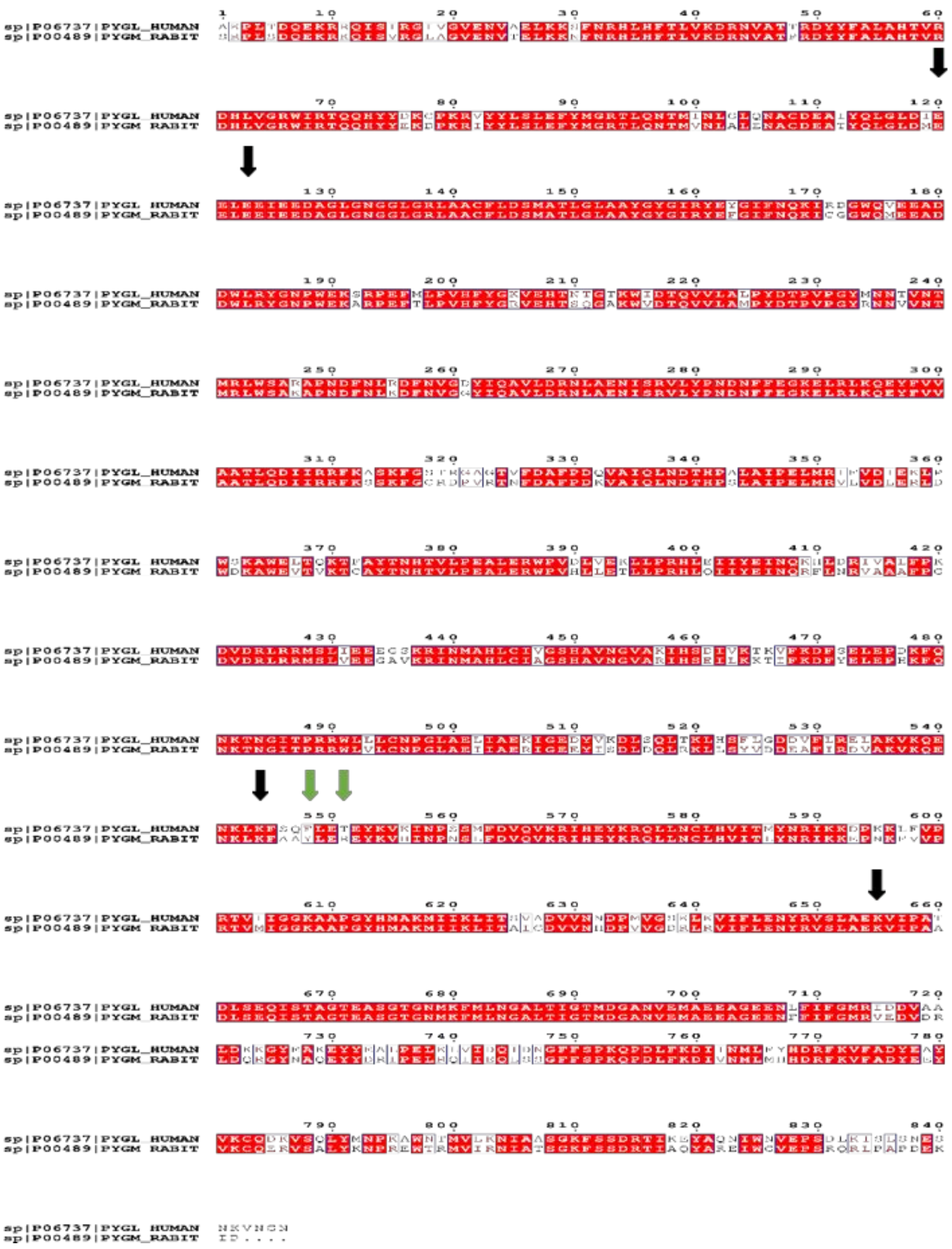

Figure S1: Sequence alignment of GP isoforms by T-coffee. The conserved amino acids of the QBS are shown with black arrows and the non-conserved amino acids 548 and 551 of the QBS are shown with the green arrows. This figure was prepared using ESPript / ENDscript bio tool.

Table S1: Summary of the diffraction, data processing and refinement statistics for the rmGPb complexes.

**Table S1.A.: Statistics of data collection, processing and refinement of the rmGPb-EGCG complex.**	
PDB id	8QMU
EMBL-Hamburg station P13; λ (Å)	0.9763
Space group	$P4_32_12$
Cell dimensions (Å), a,b,c, α,β,γ	*126.86, 126.86, 115.43,*<br>*90.00, 90.00, 90.00*
Resolution (Å)	126.86-2.00
Outermost shell (Å)	2.05-2.00
Reflections measured	701278 (50931)
Unique reflections (σ>0)	64044 (4451)
Rsymma	0.134 (0.573)
Completeness %	100 (99.9)
<I/σI>	12 (5.3)
Multiplicity	10.9 (11.4)
$CC^{1/2}$	0.994 (0.975)
Wilson Plot B-value ($Å^2$)	35.61
Reflections used for refinement	60713
Final Rcrystb (Rfreec) %	16.60 (20.84)
No of water molecules	606
No of EGCG atoms	51
r.m.s. deviation from ideality	
in bond lengths (Å)	0.0094
in bond angles (^{o})	1.5354
Average B factor ($Å^2$)	
Protein atoms	36.73
Water molecules	44.2
DMSO	76.23
Inhibitor atoms	51.03
Ramachandran (u–w) plot	Residues in most favored regions 96.14%. Residues in allowed regions 2.86%
Values in parentheses are for the outermost shell.<br>aRsymm = $\Sigma_h\Sigma i|I(h)-Ii(h)/\Sigma h\Sigma iIi(h)$ where Ii(h) and I(h) are the ith and the mean measurements of the intensity of reflection h. bRcryst = $\Sigma h|F_o-F_c|/\Sigma hF_o$, where F_o andF_c are the observed and calculated structure factors amplitudes of reflection h, respectively. cRfree is equal to Rcryst for a randomly selected 5% subset of reflections not used in the refinement.	

**Table S1.B.: Statistics of data collection, processing and refinement of the rmGPb-EGCG-Glucose complex.**	
PDB id	8R53
EMBL-Hamburg station P13; λ (Å)	0.9763
Space group	$P4_32_12$

Cell dimensions (Å), a,b,c, α,β,γ	*126.18, 126.18, 115.23,*<br>*90.00, 90.00, 90.00*
Resolution (Å)	126.18-2.00
Outermost shell (Å)	2.05-2.00
Reflections measured	688621 (47906)
Unique reflections (σ>0)	63292 (4389)
Rsymma	0.051 (0.166)
Completeness %	100 (100)
<I/σI>	27 (10.8)
Multiplicity	10.9 (10.9)
$CC^{1/2}$	0.999 (0.995)
Wilson Plot B-value ($Å^2$)	28.7
Reflections used for refinement	60075
Final Rcrystb (Rfreec) %	15.72 (19.73)
No of water molecules	821
No of EGCG atoms	51
No of glucose atoms	24
r.m.s. deviation from ideality	
in bond lengths (Å)	0.0103
in bond angles (^{o})	1.5871
Average B factor ($Å^2$)	
Protein atoms	29.11
Water molecules	38.29
DMSO	63.8
Inhibitor atoms	50.68
Ramachandran (u–w) plot	Residues in most favored regions 95.75%. Residues in allowed regions 3.37%
Values in parentheses are for the outermost shell.<br>aRsymm = $\Sigma_h\Sigma i$|I(h)-Ii(h)/ΣhΣiIi(h) where Ii(h) and I(h) are the ith and the mean measurements of the intensity of reflection h. bRcryst = Σh|F_o-F_c|/ΣhF_o, where F_o andF_c are the observed and calculated structure factors amplitudes of reflection h, respectively. cRfree is equal to Rcryst for a randomly selected 5% subset of reflections not used in the refinement.	

**Table S1.C.: Statistics of data collection, processing and refinement of the rmGPb-EGCG-Caffeine complex.**	
PDB id	8R6V
EMBL-Hamburg station P13; λ (Å)	0.9763
Space group	$P4_32_12$
Cell dimensions (Å), a,b,c α,β,γ	*126.44, 126.44, 115.35,*<br>*90.00, 90.00, 90.00*
Resolution (Å)	126.44-2.50
Outermost shell (Å)	2.60-2.50
Reflections measured	349991 (38034)
Unique reflections (σ>0)	32975 (3649)
Rsymm**a**	0.082 (0.198)
Completeness %	100 (100)
<I/σI>	18.6 (9.4)

Multiplicity	10.6 (10.4)
$CC^{1/2}$	0.998 (0.986)
Wilson Plot B-value ($Å^2$)	25.94
Reflections used for refinement	31357
Final Rcrystb (Rfree**c**) %	16.49 (23.34)
No of water molecules	474
No of EGCG atoms	51
No of caffeine atoms	24
r.m.s. deviation from ideality	
in bond lengths (Å)	0.0144
in bond angles (^{o})	1.5898
Average B factor ($Å^2$)	
Protein atoms	24.46
Water molecules	34.68
DMSO	76.49
Inhibitor atoms	60.34
Ramachandran (u–w) plot	Residues in most favored regions 95.11%Residues in allowed regions 4.14%

Values in parentheses are for the outermost shell.
aRsymm = Σ_hΣi|I(h)-Ii(h)/ΣhΣiIi(h) where Ii(h) and I(h) are the ith and the mean measurements of the intensity of reflection h. bRcryst = Σh|F_o-F_c|/ΣhF_o, where F_o andF_c are the observed and calculated structure factors amplitudes of reflection h, respectively. cRfree is equal to Rcryst for a randomly selected 5% subset of reflections not used in the refinement.

**Table S1.D.: Statistics of data collection, processing and refinement of the rmGPb-EGC complex.**	
PDB id	8R52
EMBL-Hamburg station P13; λ (Å)	0.9763
Space group	$P4_32_12$
Cell dimensions (Å), a,b,c α,β,γ	*125.97,125.97,115.17,*<br>*90.00, 90.00, 90.00*
Resolution (Å)	125.97-2.10
Outermost shell (Å)	2.16-2.10
Reflections measured	409503 (29585)
Unique reflections (σ>0)	48166 (3979)
Rsymm**a**	0.058 (0.204)
Completeness %	89 (91.2)
<I/σI>	20.9 (8.0)
Multiplicity	8.5.(9.4)
$CC^{1/2}$	0.998 (0.981)
Wilson Plot B-value ($Å^2$)	30.59
Reflections used fo refinement	45736
Final Rcryst**b** (Rfree**c**) %	15.37 (20.33)
No of water molecules	619
No of EGC atoms	36
r.m.s. deviation from ideality	
in bond lengths (Å)	0.0091

in bond angles (^{o})	1.5373
Average B factor (Å^2)	
Protein atoms	31.46
Water molecules	38.34
DMSO	66.48
Inhibitor atoms	58.16
Ramachandran (u–w) plot	Residues in most favored regions 96.73%.Residues in allowed regions 2.39%
Values in parentheses are for the outermost shell.<br>aRsymm = Σ_hΣi|I(h)-Ii(h)/ΣhΣiIi(h) where Ii(h) and I(h) are the ith and the mean measurements of the intensity of reflection h. bRcryst = Σh|F_o-F_c|/ΣhF_o, where F_o andF_c are the observed and calculated structure factors amplitudes of reflection h, respectively. cRfree is equal to Rcryst for a randomly selected 5% subset of reflections not used in the refinement.	

Table S2: Hydrogen bonding and van der Waals interactions of the solved rmGPb-EGCG **(A)**, rmGPb-EGC **(B)**, rmGPb-EGCG-glucose **(C)** and rmGPb-EGCG-caffeine **(D)** complexes.

S2.A. Hydrogen bonds interactions (EGCG-rmGPb complex)

**EGCG atom**	**rmGPb atom**	**Distance (Å)**
O1	Arg551 (NH2)	3.2
O1	Glu552 (OE2)	2.66
O02	Water-35	2.93
O02	Water-1037	2.51
O03	Water-1134	2.58
O7	Arg551 (NH2)	3.23
O37	Tyr548 (N)	3.3
O44	Glu120 (OE2)	2.66

Van der Waals interactions: 107

polar/polar: 14, polar/non polar: 54, non polar/non polar: 39

S2.B. Hydrogen bonds interactions (EGC-rmGPb complex)

EGC atom	rmGPb atom	Distance (Å)
O1	Glu124 (OE2)	3.27
O17	Arg551 (NH2)	3.19
O17	Water-1876	2.37
O19	Water-1037	2.55
O19	Water-1847	3.25
O19	Water-1848	2.4
O20	Glu552 (OE2)	2.71
O21	Water-1103	3.25
**Van der Waals interactions: 85**		
polar/polar: 7, polar/non polar: 41, non polar/non polar: 37		

S2.C. Hydrogen bonds interactions (EGCG-glucose-rmGPb complex)

EGCG atom	rmGPb atom	Distance (Å)
O1	Arg551 (NH2)	3.27
O1	Glu552 (OE1)	3.19
O7	Arg551 (NH2)	2.37
O02	Water-161	2.55
O02	Water-1037	3.25
O03	Water-1134	2.4
O37	Tyr548 (N)	2.71

O44	Glu120 (OE2)	3.25
**Van der Waals interactions: 93**		
polar/polar: 8, polar/non polar: 51, non polar/non polar: 34		

S2.D. Hydrogen bonds interactions (EGCG-caffeine-rmGPb complex)

EGCG atom	rmGPb atom	Distance (Å)
O1	Glu552 (OE2)	2.62
O37	Tyr548 (N)	3.24
O37	Arg551 (NH1)	3.07
O37	Arg551 (NH2)	2.55
O44	Glu120 (OE2)	3.15
**Van der Waals interactions: 99**		
polar/polar: 17, polar/non polar: 48, non polar/non polar: 34		

Table S3. Protein-ligand hydrogen bonding interactions of the predicted EGCG-hlGPa complex (top-ranked pose) from Glide-SP docking calculations (Figure 8A).

S3. Hydrogen bonds interactions (EGCG-hlGPa complex)

EGCG atom	hlGPa atom	Distance (Å)
O1	Asp118 (OD1)	2.64
O02	Glu552 (OE1)	2.6
O7	Asp118 (OD1)	2.62
O37	Lys544 (NZ)	2.98
O44	Gln96 (NE2)	2.82
O44	Glu123 (OE2)	2.79
O47	Gln96 (NE2)	3.15

Van der Waals interactions: 64

polar/polar: 13, polar/non polar: 36, non polar/non polar: 15

Table S4: EGCG, EGC, Glucose and Caffeine interactions with glycogen phosphorylase residues in the crystal.

S4.A. Van der Waals interactions (EGCG-rmGPb complex)

EGCG atom	rmGPb atom	Number of contacts
O1	Water-1103; Arg551 (CZ); Glu552 (CD);	3
C3	Arg551 (CZ); Arg551 (NH1); Arg551 (NH2); Glu124 (CG); Glu552 (OE2);	5
C6	Arg551 (CZ); Arg551 (NH1); Arg551 (NH2); Glu124 (CG);	4
O7	Glu121 (CA); Glu121 (CB); Glu121 (CG); Water-1103;	4
C9	Arg551 (NH1); Arg551 (NH2); Glu124 (CG);	3
O10	Glu120 (CG); Glu120 (CD); Glu120 (C); Glu121 (N); Glu120 (OE2); Glu121 (CA); Glu121 (CB);	7
C12	Glu120 (OE2); Arg551 (NH1); Glu124 (CG);	3
C14	Arg551 (NH1); Glu124 (CG); Glu124 (CD);	3
C15	Lys655 (CE); Glu124 (CD);	2
C20	Lys544 (CE); Lys655 (CE);	2
C21	Lys544 (CE); Water-35; DMSO-1799 (C1);	3
C24	Lys544 (CE); Water-1037; Water-35; Lys655 (CG);	4
C26	Water-1037; Water-142; Water-1134; Lys655 (CG); Lys655 (CA); Lys655 (CB);	6
C29	Lys544 (CG); Water-1134; Lys655 (CG); Lys655 (CB);	4
C31	Lys544 (CG); Lys655 (CD); Lys655 (CE);	3
C33	Tyr548 (CB); Tyr548 (CD1);	2
C36	Lys544 (O); Arg551 (CD); Arg551 (NH1);	3
C38	Lys544 (CG); Arg551 (NH1);	2
C39	Ala547 (CB); Arg551 (NH1);	2

C41	Lys544 (CG); Lys544 (CD); Glu120 (OE2); Lys544 (CE);	4
C01	Lys544 (O); Tyr548 (CB); Tyr548 (CG); Tyr548 (CD1);	4
O01	Glu124 (OE2); Lys655 (CE); Glu124 (CD);	3
O02	DMSO-1799 (S); DMSO-1799 (O); DMSO-1799 (C1)	3
O03	Lys544 (CG); Lys544 (C); Lys544 (O); Phe545 (N); Phe545 (CA); Phe545 (CB); Phe545 (CD1); Lys655 (CB);	8
C4	Arg551 (CZ); Arg551 (NH1); Arg551 (NH2); Glu124 (CG); Glu552 (OE2);	5
O35	Arg551 (NH1);	1
O37	Lys544 (O); Ala547 (CB); Ala547 (C); Arg551 (CD); Arg551 (NH1); Tyr548 (CA); Tyr548 (CB);	7
C43	Glu120 (OE2); Lys544 (CE);	2
O44	Glu120 (CD); Glu120 (OE1); Lys544 (CE);	3
C49	Water-1609	1
C50	Water-1609	1
**Total**		**107**

S4.B.Van der Waals interactions (EGCG-glucose-rmGPb complex)

EGCG atom	rmGPb atom	Number of contacts
O1	Arg551 (NE); Arg551 (CZ); Glu552 (CD);	3
C3	Arg551 (NE); Arg551 (CZ); Arg551 (NH1); Arg551 (NH2); Glu124 (CG); Glu552 (OE1);	6
C6	Arg551 (CZ); Arg551 (NH1);Arg551 (NH2); Glu124 (CG);	4
O7	Glu121 (CA); Glu121 (CB); Glu121 (CG); Arg551 (CZ); Water-1103;	5
C9	Arg551 (NH1); Glu124 (CG);	2
O10	Glu121 (N); Glu120 (OE2); Glu121 (CA); Glu121 (CB); Glu120 (CG); Glu120 (C);	6
C12	Glu120 (OE2); Glu124 (CG);	2
C14	Glu124 (CG); Glu124 (CD);	2

C15	Lys655 (CE);	1
C20	Lys544 (CE); Lys655 (CE)’	2
C21	Water-1430; Lys544 (CE); Water-161;	3
C24	Lys544 (CE); Water-1037; Water-161	3
C26	Lys655 (CA); Lys655 (CB); Lys655 (CG); Water-1134; Water-1037;	5
C29	Lys544 (CG; Water-1134; Lys655 (CB); Lys655 (CG);	4
C31	Lys544 (CG); Lys655 (CD); Lys655 (CE);	3
C33	Tyr548 (CB); Tyr548 (CD1);	2
C36	Arg551 (NH1); Lys544 (O); Arg551 (CD);	3
C38	Arg551 NH1;	1
C39	Ala547 CB; Arg551 NH1;	2
C41	Lys544 CG; Lys544 CD; Glu120 OE2; Lys544 CE;	4
C01	Lys544 O; Tyr548 CB; Tyr548 CG; Tyr548 CD1;	4
O01	Glu124 OE2; Lys655 CE; Glu124 CD;	3
O02	DMSO-1798 (C1);	1
O03	Lys544 CG; Lys544 C; Lys544 O; Phe545 N; Phe545 CA; Phe545 CB; Phe545 CD1; Lys655 CB;	8
C4	Arg551 CZ; Arg551 NE; Glu124 CG; Glu552 OE1;	4
O37	Ala547 (CB); Lys544 (O); Ala547 (C); Tyr548 (CA); Tyr548 (CB); Arg551 (CD);	6
C43	Glu120 OE2;	1
O44	Glu120 CD; Lys544 CE;	2
C49	Water-112	1
**Total**		**93**

S4.C. Van der Waals interactions (EGCG-glucose-rmGPb complex)

Glucose atom	rmGPb atom	Number of contacts
C1	Leu136 (N); Water-1593; Water-1019; His377 (CB); His377 (O); Water-29;	6
O1	Gly135 (CA); Gly135 (C); Leu136 (N); Water-1461; Gly135 (N);	5

O5	Gly135 (C); Leu136 (N); Leu136 (CA); Leu136 (CB); His377 (CB); His377 (CG); His377 (ND1);	7
C5	Gly135 (CA); Water-1353; Gly135 (C); Gly135 (O); Leu136 (N);	5
C6	Leu139 (CD2); Asn484 (ND2); Gly135 (C); Gly135 (O); Leu136 (N); Leu136 (CA); His377 (ND1);	7
O6	Leu139 (CD2); Asn484 (CG); Asn484 (OD1); Val455 (CB); Val455 (CG1); His377 (CG); His377 (CE1); Val455 (CG2);	8
C4	Gly675 (N); Asn484 (ND2); Water-1353;	3
O4	Ser674 (CB); Ser674 (C); Gly675 (CA); Gly675 (C); Gly675 (O);	5
C3	Gly675 (N); Water-1353; Glu672 (OE2); Water-1593; Water-1388;	5
O3	Ser674 (C); Gly675 (CA); Glu672 (CG); Glu672 (CD); Ala673 (N); Ala673 (CA); Ala673 (CB); Water-1388;	8
C2	Glu672 (OE2); Water-1593; His377 (O); Water-29; Water-1388;	5
O2	His377 (O); Asn284 (CG);	2
**Total**		**66**

S4.D.Van der Waals interactions (EGCG-caffeine-rmGPb complex)

EGCG atom	rmGPb atom	Number of contacts
O1	Arg551 (NE); Arg551 (CD); Glu552 (CD);	3
C3	Arg551 (NE); Arg551 (CZ); Arg551 (NH2); Glu124 (CG); Glu552 (OE2);	5
C6	Glu124 (CG);	1
O7	Glu121 (CA); Glu121 (CB); Glu121 (CG); Water-1103;	4
C9	Glu120 (OE2); Glu124 (CG);	2
O10	Glu120 (OE2); Glu120 (C); Glu121 (N); Glu121 (CA); Glu121 (CG);	5
C12	Glu120 (OE2); Glu124 (CG);	2
C14	Glu124 (CG); Glu124 (CD);Arg551 (NH2);	3
C15	Arg551 (NH2); Lys655 (CE);	2
C20	Lys655 (CE);	1
C21	Water-1430;	1

C24	Lys655 (CG);	1
C26	Lys544(CD); Lys655 (CA); Water-1134; Lys655 (CG);	4
C29	Lys544 (CG); Lys544 (CD); Lys655 (CB); Water-1134; Lys655 (CG);	5
C31	Lys544 (CG); Lys544 (CD); Lys655 (CG);	3
C33	Tyr548 (CB); Tyr548 (CD1); Arg551 (NH2)	3
C36	Lys544(O); Arg551 (CZ); Arg551 (NH1); Arg551 (NH2)	4
C38	Lys544 (CG)	1
C39	Ala547 CB; Water-1609;	2
C41	Lys544 (CG); Glu120 (OE2); Lys544 (CE);	3
C01	Lys544 (CG); Lys544 (O); Tyr548 (CB); Tyr548 (CG); Tyr548 (CD1);	5
O01	Glu120 (OE2); Glu124 (OE2); Lys655 (CE); Water-1430; Glu124 (CE);	5
O02	Water-1618; DMSO-923 (S); DMSO-923 (O); DMSO-923 (C1);	4
O03	Lys544 (CB); Lys544 (CG); Lys544 (CD); Lys544 (C); Lys544 (O); Phe545 (N); Water-1134; Phe545 (CA);	8
C4	Arg551 (NE); Arg551 (CZ); Arg551 (NH2); Glu552 (OE2); Glu124 (CG); Glu124 (CD); Glu124 (OE1);	7
O35	Lys544 (CG); Arg551 (NH2);	2
O37	Lys544 (O); Ala547 (CB); Ala547 (C); Tyr548 (CA); Arg551 (CZ);Tyr548 (CB);	6
C43	Glu120 OE2;	1
O44	Glu120 (OE1); Glu120 CD; Lys544 CE;	3
C46	Water-1609	1
C49	Water-1609	1
C50	Water-1609	1
**Total**		**99**

S4.E. Van der Waals interactions (EGCG-caffeine-rmGPb complex)

Caffeine atom	rmGPb atom	Number of contacts

N1	Tyr613 (CG); Tyr613 (CD1); Tyr613 (CE1); Phe285 (CD1); Phe285 (CE1); Phe285 (CZ)	6
C2	Tyr613 (CG); Tyr613 (CD1); Tyr613 (CE1); Tyr613 (CE2); Tyr613 (CZ); Phe285 (CD1); Water-1814; Phe285 (CE1);	8
C10	Tyr613 (CE1); Phe285 (CE1);Water-1795;	3
C6	Tyr613 (CG); Tyr613 (CD1); Tyr613 (CB); Phe285 (CD1); Phe285 (CE1); Phe285 (CE2); Phe285 (CZ);	7
N3	Tyr613 (CG);Tyr613 (CD1);Tyr613 (CD2);Tyr613 (CE1); Tyr613 (CE2); Tyr613 (CZ); Phe285 (CB); Phe285 (CG); Phe285 (CD1); Phe285 (CE1);	10
O11	Tyr613 (CE1); Tyr613 (CZ);Tyr613 (OH);	3
C12	His571 (ND1); His571 (CE1); Tyr613 (CD2); Tyr613 (CE2); Tyr613 (CZ); Phe285 (CB); Phe285 (CD1); Water-1814;	8
C4	Water-1128; Tyr613 (CG); Tyr613 (CD1); Tyr613 (CD2); Tyr613 (CB); Phe285 (CB); Phe285 (CG); Phe285 (CD1); Phe285 (CD2); Phe285 (CE1);	10
C5	Tyr613 (CG); Tyr613 (CD1); Tyr613 (CA); Tyr613 (CB); Phe285 (CG); Phe285 (CD1); Phe285 (CD2); Phe285 (CE1); Phe285 (CE2); Phe285 (CZ);	10
N9	Tyr613 (CG); Ty613 (CD2); Ala610 (CB); Tyr613 (CB); Phe285 (CB); Phe285 (CG); Phe285 (CD1); Phe285 (CD2);	8
O13	Gly612 (O); Phe285 (CE1); Phe285 (CE2); Phe285 (CZ); Water-1796;	5
N7	Asn282 (OD1); Ala610 (CB); Tyr613 (N); Tyr613 (CA); Tyr613 (CB); Phe285 (CG); Phe285 (CD2); Phe285 (CE2);	8
C8	Asn282 (OD1); Water-1128; Ala610 (CB); Tyr613 (CB); Phe285 (CB); Phe285 (CG); Phe285 (CD2);	7
C14	Asn282 (OD1); Gly612 (CA); Gly612 (C); Tyr613 (N); Tyr613 (CA); Water-1561; Gly612 (O); Phe285 (CD2); Phe285 (CE2); DMSO-927 (O);	10
**Total**		**103**

S4.F. Van der Waals interactions (EGC-rmGPb complex)

EGC atom	rmGPb atom	Number of contacts
O1	Glu124 (CD); Lys655 (CE);	2
C2	Glu124 (OE2); Tyr548 (CD1); Lys655 (CE);	3
C3	Arg551 (CZ); Arg551 (NH2); Tyr548 (CB); Tyr548 (CD1); Arg551 (NE); Water-1876;	6
C4	Lys544 (O); Tyr548 (CB); Tyr548 (CG); Tyr548 (CD1); Water-1876;	5
C5	Lys544 (CG); Lys544 (CE); Lys655 (CG); Lys655 (CD); Lys655 (CE);	5
C6	Lys544 (CG); Lys544 (CD); Lys544 (CE); Lys655 (CB); Lys655 (CG);	5
C7	Lys544 (CD); Lys544 (NZ); Lys655 (CA); Lys655 (CB); Water-1037; Lys655 (CG);	6
C8	Lys544 (NZ); Water-1037; Lys655 (CG); Water-1848;	4
C9	Lys544 (CE); Lys544 (NZ); Lys655 (CE); Water-1848;	4
C10	Lys544 (CE); Lys544 (NZ); Lys655 (CD); Lys655 (CE);	4
C11	Glu124 (CG); Glu124 (CD); Glu124 (OE2);	3
C12	Arg551 (NH1); Glu124 (CG); Glu124 (CD); Arg551 (CD); Glu552 (OE2);	5
C13	Arg551 (NH1); Glu124 (CG); Glu552 (OE2);	3
C14	Arg551 (NH1); Glu124 (CG);	2
C15	Arg551 (NH1); Glu124 (CG);	2
C16	Glu124 (CG);	1
O17	Arg551 (CZ); Lys544 (CG); Lys544 (CE); Arg551 (NE);	4
O18	Lys544 (CB); Lys544 (CG); Lys544 (C); Lys544 (O); Phe545 (N); Phe545 (CA); Phe545 (CB); Lys655 (CB);	8
O19	Water-1618; Lys544 (NZ);	2
O20	Glu552 (CD);	1
O21	Glu121 (CB); Glu121 (CG); Glu121 (CD); Glu121 (CA);	4
O22	Glu121 (N); Glu121 (CB); Glu121 (CG); Glu120 (C); Glu120 (O); Glu121 (CA);	6
**Total**		**85**

S4.G. Van der Waals interactions (EGCG-hlGPa complex)

EGCG atom	rmGPb atom	Number of contacts
C24	Glu552 (OE1);	1
C26	Glu552 (OE1); Phe548 (CE1); Phe548 (CD1);	3
C29	Phe548 (CE1); Phe548 (CD1);	2
C01	Lys655 (CE); Glu124 (CD); Glu124 (CG); Glu124 (OE2);	4
C33	Glu124 (CG); Glu124 (OE2);	2
C12	Glu120 (OE1);	1
C9	Glu120 (OE1); Glu120 (CD);	2
C6	Asp118 (OD1);	1
C3	Asp118 (OD1);	1
O02	Glu552 (CD);Thr551 (OG1);	2
O03	Phe548 (CE1); Phe548 (CD1); Lys655 (CE); Glu124 (CD); Glu124 (OE1); Glu124 (OE2);	6
O35	Glu120 (OE1); Glu124 (OE2);	2
O1	Glu121 (OE1) ; Glu121 (CG); Glu121 (CD); Asp118 (CG);	4
O7	Asp118 (CG); Asp118 (OD2);	2
O10	Lys544 (CE); Glu120 (OE1); Glu120 (CD); Glu120 (OE2);	4
C38	Lys655 (CE); Lys544 (NZ); Glu120 (OE1);	3
C36	Lys544 (CE); Lys544 (NZ); Glu120 (OE1);	3
O37	Lys544 (CE); Glu120 (OE1);	2
C41	Glu120 (OE1); Glu124 (OE2); Glu120 (CD);	3
C43	Glu120 (OE1); Gln96 (NE2);	2
C46	Gln96 (NE2);	1
C49	Lys655 (CG); Lys544 (NZ);	2
C39	Lys655 (CG); Lys655 (CE); Lys544 (NZ);	3
O50	Lys655 (CG); Glu654 (C); Glu654 (O);	3
O47	Leu494 (CD2); Leu494 (O);Gln96 (OE1); Gln96 (CD);	4
O44	Glu123 (CD);	1
**Total**		**64**

S4.H. Hydrogen bonds interactions (EGCG-glucose-rmGPb complex)

Glucose atom	rmGPb atom	Distance (Å)
O1	Water-1593	3.01
O1	Water-1019	2.87
O2	Glu672 (OE2)	3.23
O2	Tyr573 (OH)	3.12
O2	Water-1593	2.79
O2	Asn284 (OD1)	3.29
O2	Asn284 (ND2)	3.1
O2	Water-29	3.3
O2	Water-1388	2.69
O3	Ser674 (N)	3.17
O3	Glu672 (OE2)	2.77
O3	Gly675 (N)	3.11
O4	Gly675 (N)	2.97
O4	Asn484 (ND2)	3.3
O4	Water-1353	2.72
O6	Asn484 (ND2)	2.71
O6	His377 (ND1)	2.71
**Van der Waals interactions: 66**		
polar/polar:11, polar/non polar: 47, non polar/non polar: 8		

S4.J. Hydrogen bonds interactions (EGCG-caffeine-rmGPb complex)

Caffeine atom	rmGPb atom	Distance (Å)
O11	Water-1814	2.83
N9	Water-1128	2.79
**Van der Waals interactions: 103**		
polar/polar: 5, polar/non polar: 47, non polar/non polar: 51		

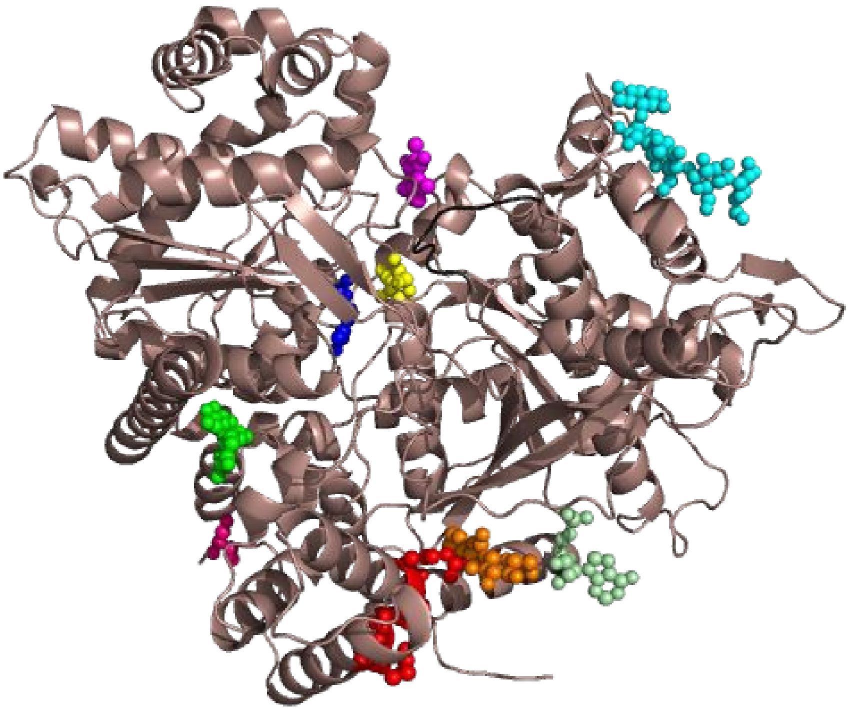

Figure S2: The catalytic site (yellow), the inhibitor site (purple), the allosteric site (palegreen), the new allosteric site (red), the glycogen storage site (light blue), the quercetin binding site (green), the benzamidazole site (orange), PLP (blue), Ser14 (pink), and the 280s loop (black) were all represented in the rmGPb monomer crystal structure.

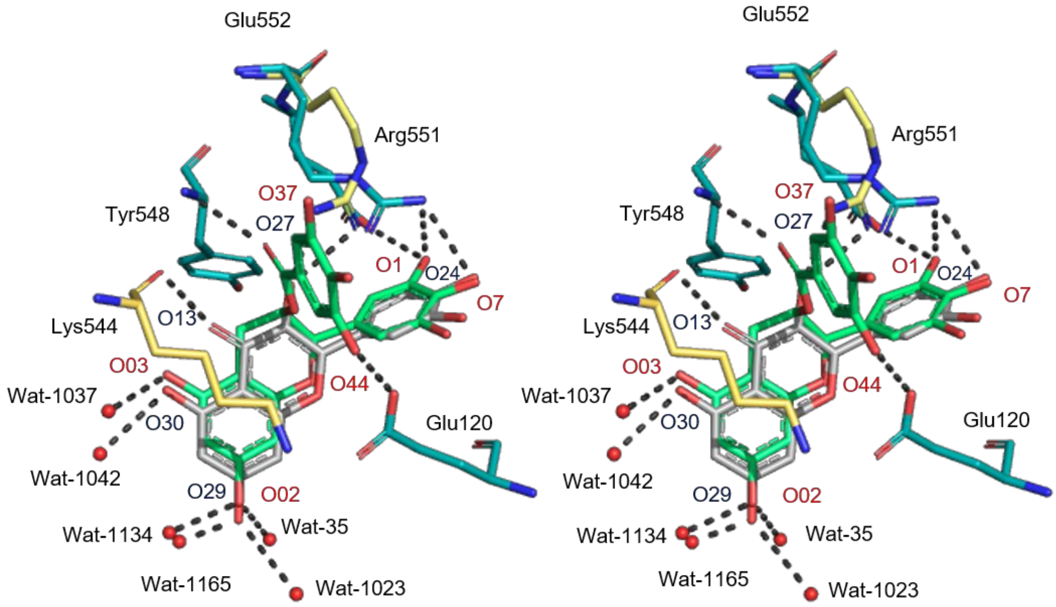

Figure S3: Stereo diagram of EGCG (green) and quercetin [(PDB entry: 4MRA), white] binding to the quercetin binding site (QBS) of rmGPb. Hydrogen bond interactions are represented by dotted lines and water molecules as spheres.

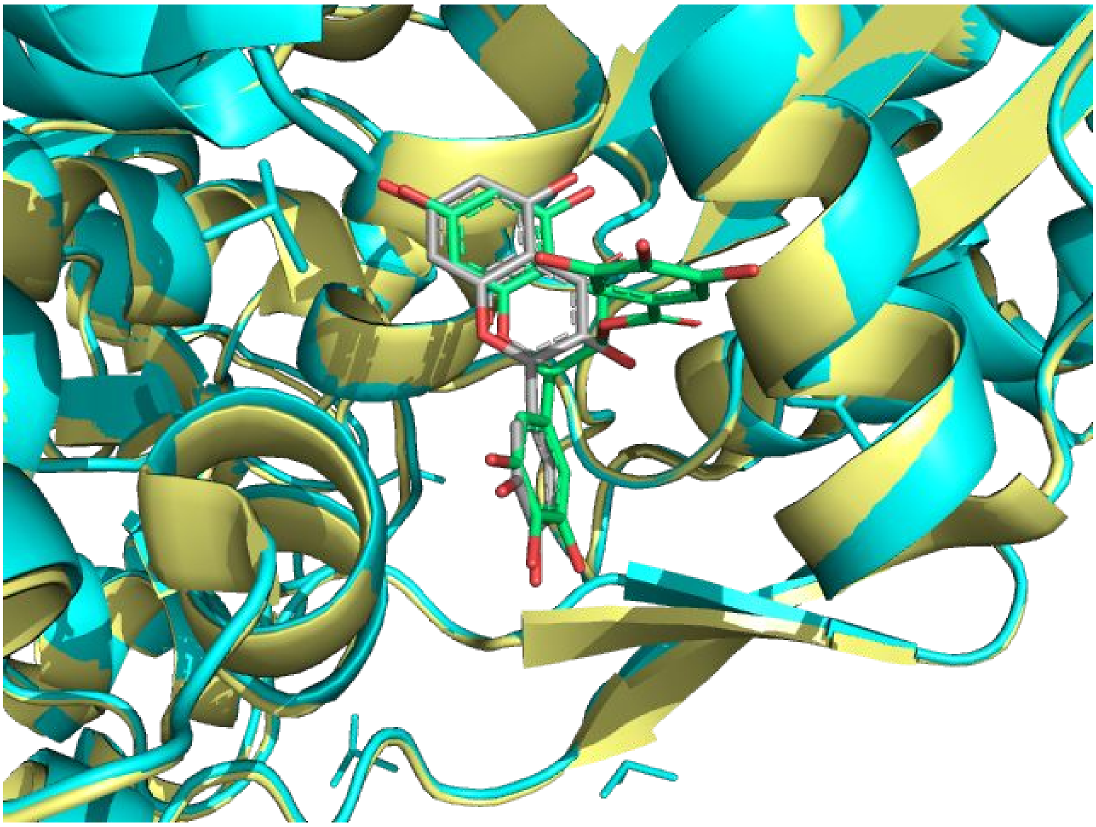

Figure S4: Superposition of EGCG (green) - rmGPb (cyan) and quercetin [(PDB entry: 4MRA) white] - rmGPb (paleyellow) complexes.
